# Sorting Nexin 17 Regulates ApoER2 Recycling and Reelin Signaling

**DOI:** 10.1371/journal.pone.0093672

**Published:** 2014-04-04

**Authors:** Pablo Sotelo, Pamela Farfán, María Luisa Benitez, Guojun Bu, María-Paz Marzolo

**Affiliations:** 1 Departamento de Biología Celular y Molecular, Facultad de Ciencias Biológicas, Millenium Nucleus for Renerative Biology (MINREB), Pontificia Universidad Católica de Chile, Santiago, Chile; 2 Department of Neuroscience, Mayo Clinic, Jacksonville, Florida, United States of America; Institute of Molecular and Cell Biology, Biopolis, United States of America

## Abstract

ApoER2 is a member of the low density-lipoprotein receptor (LDL-R) family. As a receptor for reelin, ApoER2 participates in neuronal migration during development as well as synaptic plasticity and survival in the adult brain. A previous yeast two-hybrid screen showed that ApoER2 is a binding partner of sorting nexin 17 (SNX17) - a cytosolic adaptor protein that regulates the trafficking of several membrane proteins in the endosomal pathway, including LRP1, P-selectin and integrins. However, no further studies have been performed to investigate the role of SNX17 in ApoER2 trafficking and function. In this study, we present evidence based on GST pull-down and inmunoprecipitation assays that the cytoplasmic NPxY endocytosis motif of ApoER2 interacts with the FERM domain of SNX17. SNX17 stimulates ApoER2 recycling in different cell lines including neurons without affecting its endocytic rate and also facilitates the transport of ApoER2 from the early endosomes to the recycling endosomes. The reduction of SNX17 was associated with accumulation of an ApoER2 carboxy-terminal fragment (CTF). In addition, in SNX17 knockdown cells, constitutive ApoER2 degradation was not modified, whereas reelin-induced ApoER2 degradation was increased, implying that SNX17 is a regulator of the receptor's half-life. Finally, in SNX17 silenced hippocampal and cortical neurons, we underscored a positive role of this endosomal protein in the development of the dendritic tree and reelin signaling. Overall, these results establish the role of SNX17 in ApoER2 trafficking and function and aid in identifying new links between endocytic trafficking and receptor signaling.

## Introduction

ApoER2 is a member of the LDL-R family that is expressed in neurons, platelets, ovaries, epididymis, placenta and brain [1], [2], [3]. Together with VLDL-R, which is another member of the LDL-R family [4], and Ephrin [5], ApoER2 binds the extracellular matrix protein reelin during development [4], [6], triggering a signaling pathway that regulates neuronal migration and the formation of laminated brain structures such as the cortex, hippocampus [7] and cerebellum [4] as well as the migration of sympathetic preganglionic neurons in the spinal cord [8], [9]. Reelin binding to ApoER2 induces binding of the adaptor protein Dab1 to its cytoplasmic NPxY motif [10], [11], resulting in the phosphorylation of specific tyrosines [12], [13], PI3K activation, and n-cofilin phosphorylation in the leading processes of migrating neurons [9], [14]. In the adult, ApoER2/reelin have roles in synaptic function, learning, and memory. This is exemplified by the phenotype of the ApoER2 knockout, which has normal baseline synaptic activity but presents deficiencies in the long-lasting forms of synaptic plasticity [15]. Both ApoER2 and VLDLR knockout mice present deficient long-term potentiation (LTP) when they are stimulated by reelin, suggesting a cooperative effect of both receptors [15]. The participation of ApoER2 in LTP is in part due to its interaction with the NMDA receptor [16], [17] and its intracellular association with PSD-95, a protein that mediates the interaction with the NMDA receptor [18]. PSD-95 interacts with ApoER2 splicing variants including exon 19, which encodes a proline-rich insert in the cytoplasmic domain of the receptor [18], [19]. ApoER2 and reelin affect dendritic development in the hippocampus by increasing the length of dendrites [20]. These proteins also play a role in the development of apical dendrites in hippocampal pyramidal neurons in the mouse brain, in a manner that is dependent on the participation of Dab1 and the Src family kinases [21]. Finally, the reelin signaling pathway has also been shown to be relevant to neuroregeneration in both the peripheral and the central nervous systems [22], [23].

ApoER2 is constitutively internalized, and this process can also be induced by its ligand, reelin. Therefore, its cell surface expression is dependent on its clathrin-mediated internalization [24] and most likely also on its recycling efficiency. Relevant to the endocytosis and recycling of ApoER2 is the adaptor protein SNX17, a member of the sorting nexin family, which contains a characteristic Phox homology (PX) as well as protein 4.1, ezrin, radixin, and moesin (FERM) domains that participate in the intracellular trafficking of membrane proteins [25]. A yeast two-hybrid screen showed that SNX17 interacts with P-selectin [26] and LDL-R family proteins [26], [27]. Its PX domain facilitates the interaction between SNX17 and PI3P-enriched membranes, particularly with the early endosome membranes that are positive for EEA1 [27]–[29]. SNX17 increases the endocytosis of P-selectin and diminishes the transport of this membrane protein to the late endosome [30]. SNX17 also increases the number of endocytic cycles performed by the LDL-R [27]. In addition, SNX17 participates in the recycling of LRP1 - another member of the LDL-R family [31] - regulates the cell surface levels and half-life of APP [32] and mediates the recycling and degradation of β1-integrins [33], [34]. SNX17, specifically through its FERM domain, recognizes an NPxY motif in the cytoplasmic domains of LRP1, APP and β1 integrins [31], [32], [34], [35]. Recently, we proposed that SNX17 also participates in the basolateral and somatodendritic recycling of LRP1 in polarized epithelial and neuronal cells respectively [36]. In the case of ApoER2, the studies by Stockinger et al that utilized a yeast two-hybrid screen, and identified SNX17 as an interacting protein using ApoER2 tail as bait [27]. However, in contrast, the authors discarded the NPxY motif as a SNX17 binding motif for the receptors - including ApoER2 - in part because megalin/LRP2, another receptor of the LDL-R family, has three NPxY motifs but did not bind SNX17 [27], [37]. No further evidence has been generated indicating a role for SNX17 in ApoER2 trafficking and function.

Similarly to APP, ApoER2 is proteolytically processed by secretases [38]. As a result of the first processing step, a soluble ectodomain fragment and a membrane-associated fragment corresponding to the carboxy-terminal fragment (CTF) of the receptor are produced [39]. When ApoER2-expressing cells are treated with DAPT, a γ–secretase inhibitor, the amount of CTFs increase [39], [40], indicating that γ–secretase is responsible for the ApoER2-CTF processing to produce the corresponding intracellular domain (ICD). On the other hand, the α-secretase protein inhibitor TIMP3 also decreases the levels of the soluble extracellular fragment and CTF of ApoER2, suggesting an active role of α-secretase in the first step of ApoER2 processing at the plasma membrane [41]. There is also evidence showing that reelin induces ApoER2 processing [42], [43] and degradation [43], however the mechanisms by which this is accomplished by reelin is not completely clear. On the other hand, it is likely that the mechanisms are related to modifications in the trafficking/cell surface expression of this receptor, as these effects also occur in cells lacking the signaling adaptor protein Dab1.

In this report, we present evidence showing that the ApoER2-SNX17 interaction depends on the cytoplasmic NPxY endocytosis/signaling motif of the receptor. SNX17 knock down (KD) was associated with an inhibition of ApoER2 recycling by inducing its retention in the Rab5-positive early endosomal compartment, and delaying its trafficking to the Rab11-positive recycling compartment. In SNX17 knockdown cells, there was an accumulation of ApoER2-CTF and the reelin induced receptor degradation was increased. These results relate the endocytic/recycling route of ApoER2 with the receptor half-life, which is regulated by reelin. Finally, the neuronal function of ApoER2 was found to be altered in SNX17 knockdown hippocampal and cortical neurons. We observed a significant decrease in the response to reelin in this system. This decrease was reflected by a partial but significant inhibition in dendritic outgrowth as well asa decrease in the signaling pathway, which was indicated by the altered phosphorylation levels of Dab1, AKT, GSK3β and the ApoER2-specific target n-cofilin. These results highlight the importance of the SNX17-regulated link between ApoER2 trafficking and its signaling properties.

## Materials and Methods

### Reagents, antibodies, and constructs

DMEM, L-glutamine, penicillin-streptomycin, and trypsin were purchased from Gibco (Life Technologies Inc., Grand Island, NY, USA). Fetal bovine serum (FBS) was purchased from Hyclone (South Logan, UT, USA). Individual protease inhibitors, puromycin, cycloheximide (CHX), and all chemical reagents were purchased from Sigma-Aldrich. The polyclonal anti-SNX17 antibody was raised against a 14-amino-acid peptide corresponding to the carboxyl-terminal region of SNX17 protein as previously described [32]. The rabbit anti-apoER2 tail was used as previously described [44], and the monoclonal anti-apoER2 antibody that recognizes the proline-rich insert in the cytoplasmic ApoER2 tail was from Sigma. The monoclonal anti-myc antibody and anti-GST were from Roche Molecular Biochemicals (Hague Road, Indianapolis, IN, USA). The chicken polyclonal anti-HA antibody, mouse monoclonal anti-EEA1, anti-GSK3β, anti-phospho-tyrosine, anti-actin antibodies, and peroxidase-labeled antibodies were all from Chemicon International (Temecula, CA, USA). Anti-Dab1 was from Millipore; Anti-phospho-GSK3β (Ser 9) and anti-βIII-tubulin antibody were from Upstate; anti-phospho-AKT (S473) was from Cell Signaling (Davers, USA). Anti-AKT was from SAB (Pearland, USA). Rabbit anti-phospho-cofilin was kindly provided by Dr. James Bamburg (Colorado State University). Anti-LAMP2 and anti-rab11 antibodies were from Zymed Laboratories (San Francisco, CA). Anti-γ–adaptin antibody was from Abcam (Abcam Inc, Cambridge, MA). All Alexa-conjugated antibodies and anti-Alexa 488 antibody were purchased from Molecular Probes (Invitrogen). The ECL System was from Pierce (Rockwell, USA). Immobilon-P transfer membranes were from Millipore (Billerica, MA, USA). The plasmids for the expression of HA-ApoER2 and GST-ApoER2 tails were described previously [24]. The mouse SNX17 cDNAs tagged at the 5′ end with a 9xmyc epitope (myc-SNX17) and the GST-SNX17 construct were described previously [31], [32]. The shRNA-containing pLKO.1 vector used to silence human SNX17 was from Open Biosystems (RHS3979-98493075), and the construct used to silence mouse SNX17 was from Sigma (TRCN0000202153).

### Cell culture

HEK 293 and HeLa cells were maintained in DMEM with 10% fetal bovine serum (FBS) (Sigma) and L-glutamine (Invitrogen). Parental N2a were grown in DMEM with 7.5% FBS. All media were supplemented with 100 U penicillin, 1 U streptomycin, and 5 μg/mL plasmocin. HA-ApoER2- and HA-ApoER2-NPxA-expressing N2a cells [24], [54] were maintained in growth medium supplemented with 400 μg/mL G418.

The protocols to obtain neurons from rat embryos were carried out with approval from the Bioethical Board for animal studies at the Facultad de Ciencias Biológicas and according to the Guide for the Care and Use of Laboratory Animals of CONICYT. Hippocampal and cortical neurons were cultured as described [45]. Dissociated cells were plated on glass coverslips coated with 1 mg/mLpoly-L-lysine in medium containing 10% horse serum (Invitrogen-BRL). After 3 h, the medium was supplemented with N2 (Invitrogen-BRL; [46]). Cells were transiently transfected using Lipofectamine or infected with lentivirus.

### Lentivirus preparation and infection

For lentivirus production, HEK 293T cells were transfected with empty pLKO.1 or the appropriate shRNA-containing pLKO.1 construct together with the pCMVR8.91 and pCMV-VSV-G packaging systems as described previously [47], [48]. Forty-eight hours after transfection, the culture supernatant was centrifuged to remove any cells and then used to infect different cell lines in the presence of 8 μg/mL polybrene. To produce cellular clones, cells were incubated with 2 μg/mL puromycin in growth medium until the control cells (without infection) died.

To infect neurons, virus supernatants from transfected HEK293 cells were collected in Optimem. The supernatant was concentrated and purified by ultrafiltration using Amicon Ultra 100K (Millipore). The resultant viral particles were stored at -80°C. Virus titer was calculated by dilution limit and resistance to puromycin colony formation.

### GST Pull-down Assay

GST-fusion proteins were prepared as described previously [24], [31]. The fusion proteins were expressed in *Escherichia coli* (BL21) and lysed with PBS, 1% Triton X-100, 10 mM EDTA, and 1X complete protease inhibitor mixture. The proteins were purified and then sequentially dialyzed against PBS, 250 mM NaCl, 50 mM Tris–HCl (pH 8.0), and 5.0 mM Tris–HCl (pH 8.0). HEK293 cells were transfected with the HA tagged- ApoER2 expressing plasmids or plasmids for myc-SNX17 constructs using the calcium phosphate method. Cell extracts were prepared by lysing the cells with HUNT buffer (20 mM Tris–HCl, pH 8.0, 100 mM NaCl, 1 mM EDTA, 0.5% Nonidet P-40, 1 mM phenylmethylsulfonyl fluoride, and 1X complete protease inhibitor mixture). The cell extracts were incubated with GST fusion proteins and bound to glutathione agarose beads for 3 h at 4°C. The beads were washed three times in HUNT buffer, boiled in 2X sample buffer, and separated by SDS-PAGE. The presence of SNX17 or ApoER2 was determined by western blot analysis using the appropriate anti-myc or anti-HA monoclonal antibody.

### Determinations using fluorescence-activated cell sorting (FACS)

FACS analyses of surface and total ApoER2 was performed as previously described [44]. Briefly, N2a cells stably expressing ApoER2 or HEK 293 clones were plated in 100-mm dishes. Silenced and pLKO HEK 293 cells were transfected with the plasmids pcDNA3-HA-ApoER2 and pcDNA3-RAP using Lipofectamine. The ApoER2-expressing cells were washed with PBS and incubated with PBS containing 1 mM EDTA for 5 min, mechanically detached, and collected by centrifugation at 700×g for 5 min. One-third of the pellet was resuspended in 160 μL PFN (PBS, 1% heat-inactivated FBS) and kept on ice (non-permeabilized); the remaining cells were permeabilized by incubating with 160 μl PFN-saponin 0.05% and gently mixed at 4°C for 10 min. The permeabilized and non-permeabilized cells were then equally divided into microcentrifuge tubes and mixed with 50 μL PFN (control samples) or 50 μL PFN containing anti-HA in the presence or absence of 0.05% saponin. After gently rocking the tubes at 4°C for 60 min, the cells were washed and resuspended in 50 μL PFN or 50 μ μL PFN-with 0.05% saponin each containing Alexa 488-conjugated donkey anti-mouse IgG (Molecular Probes). After a 1-h incubation period, the cells were incubated with secondary antibody at 4°C, washed, and resuspended in 300 μL PFN for FACS determination in a FACS Calibur cytometer (Beckton & Dickinson). The surface and total ApoER2 fluorescence was represented as the mean of fluorescence intensity from non-permeabilized and permeabilized cells, respectively, after subtracting the corresponding blank controls. The results are plotted as % of the control shRNA. To examine phenotype recovery after SNX17 shRNA treatment, HEK 293 cell silenced clones were cotransfected with HA-ApoER2, RAP, and either full length myc-SNX17, myc-SNX17 2-250, myc-SNX17 105–470 or empty vector. Forty-eight hours later, the cells were incubated with PBS-EDTA and mechanically detached. In the case of non-permeabilized conditions, cells were washed with PFN and incubated with a chicken anti-HA antibody in PFN for 1 h at 4°C; then, cells were fixed with 4% PFA in PFN for 20 min at 4°C, permeabilized with saponin, and incubated with anti-myc antibody in PFN saponin for 1 h at 4°C. For the permeabilized condition, the cells were fixed as previously described, permeabilized with saponin, and incubated with a mouse anti-myc and a chicken anti-HA antibody for 1 h at 4°C. In both conditions, cells were then incubated with secondary antibodies and analyzed by FACS as previously described.

Endocytosis analysis by FACS was conducted as previously described [49]. N2a cell clones or HEK 293 clones transfected with the expression plasmids for HA-ApoER2 and RAP were trypsinized, and 3×10^4^ cells were plated in 12-well dishes. Twenty-four hours later, the cells were incubated with 1 μg/mL anti-HA antibody labeled with Alexa 488 in binding buffer (DMEM, 10 mM HEPES, 5 mg/mL BSA) for 1 h at 4°C. Cells were shifted to 37°C during the indicated time period to allow for receptor internalization; the cells were incubated with PBS-EDTA and washed with PFN. The remaining antibody on the surface was removed by an acid wash by incubating the cells with stripping buffer (0.1 M glycine, 0.1 M NaCl, pH 3) for 5 min. As a control for the total surface-bound antibody, cells without the acid wash were analyzed. All conditions were analyzed by FACS. The endocytic rate was calculated by subtracting the value of the cells exposed to the acid wash at time 0 (A_0_) from each time point, and dividing by A_0._

Determination of ApoER2 recycling by FACS was performed as described previously [31]. Briefly, N2a cell clones or HEK 293 clones transfected with HA-ApoER2 and RAP were trypsinized, and 3×10^4^ cells were plated in 12-well plates. The day after plating, the cells were incubated with 1 μg/mL anti-HA antibody labeled with Alexa 488 in binding buffer (DMEM, 10 mM HEPES, 5 mg/mL BSA) for 30 min at 37°C to allow for receptor internalization and trafficking. Cells were washed twice with binding buffer, and the recycled receptors were chased at the surface with a quenching anti-Alexa 488 antibody at the indicated times. As a control for the fluorescence remaining for each time point, cells were incubated in binding buffer during the chase time (non-chased value). Cells were analyzed by cell cytometry and the percentage of the initial fluorescence remaining at each time point was calculated as the difference between time 0 and each chased time point. Every time point (including time 0) was normalized to the non-chased value. The percentage of recycling efficiency was calculated by subtracting the percentage of internal fluorescence from 100.

All FACS data analyses were conducted using the Weasel program and analyzed using GraphPad 4.

### Subcellular fractionation

Subcellular fractionation was performed as previously described [50]. Briefly, 3×10^6^ N2a cell clones were plated in 100-mm plates (four plates for each condition), and 24 h later the cells were lysed mechanically using a glass dounce homogenizer. The post-nuclear supernatant (PNS) was prepared by centrifugation for 15 min at 1500 g. The PNS was adjusted to 40.2% sucrose and loaded in the bottom of a Tst 60.4 tube. Then, 1.5 mL of 35% sucrose solution and 1 mL of 25% of sucrose solution were added sequentially, followed by homogenization buffer (250 mM sucrose, 3 mM imidazole, pH 7.4) to fill the rest of the tube. The samples were centrifuged for 1 h at 34,000 rpm using a Tst 60.4 rotor. Early and recycling endosomes were collected in the 25%/35% interface and late endosomes in the top 25%. Fractions were precipitated by the methanol/chloroform method as previously described [51] and were resolved in Tris/Tricine gels.

### Colocalization analysis

HeLa cells stably expressing pLKO or shRNA against SNX17 were transfected with HA-ApoER2, RAP and GFP-Rab5, GFP-Rab7, or GFP-Rab11 using the Lipofectamine protocol; 48 h later, the cells were incubated with anti-HA antibody for 2 h at 4°C. After that, the cells were incubated at 37°C for the corresponding time to allow for internalization. The cells were washed twice with PBS, and the remaining surface antibody was removed by acid wash by incubating the cells in stripping buffer (0.1 M glycine, 0.1 M NaCl, pH 3) for 5 min. Cells were washed twice with PBS, fixed with 4% paraformaldehyde for 20 min, and permeabilized with 0.1% Triton X-100 in PBS. Intracellular localization of the receptor was detected with goat anti-mouse Alexa-594 antibody, and confocal microscopy was performed using a laser scanning LSM 510 Zeiss microscope and a 63 X oil immersion lens (numerical aperture: 1.4). The images were deconvolved using the nearest neighbor algorithm of Methamorph version 6.0r1 software (Molecular Devices). Colocalization was quantified using the JACoP plugin of the ImageJ software (http://rsb.info.nih.gov/ij/plugins/track/jacop.htlm). For each individual condition (n = 10 cells per condition), a statistical analysis of the correlation of the fluorescence signal of green and red pixels in the dual-channel image was performed using Pearson's and Mander's coefficients and the Van Steensel's approach. The amount of total fluorescent signal in the red channel that overlapped with the total fluorescent signal in the green channel was shown in the graphic.

### Analysis of surface and total ApoER2 in neurons

Surface/total analysis was performed as described previously [36]. Briefly, 1×10^5^ mouse dissociated cortical neurons were transfected at DIV 5 with HA-ApoER2, RAP, and the corresponding shRNA plasmid (0.2 μg DNA each) using the Lipofectamine method. Forty-eight hours later, the cells were fixed with 4% PFA and 4% sucrose for 20 min at 37°C. For cell surface staining, the cells were incubated for 30 min with a mouse anti-HA antibody and a rabbit anti-ApoER2 tail antibody that recognizes the intracellular tail (to identify non-specific permeabilization), and the surface attached antibodies were fixed with 4% PFA and 4% sucrose. Next, cells were washed, permeabilized, and incubated with a chicken anti-HA antibody to identify the intracellularApoER2. The cells were incubated with secondary antibodies, and stained cells were observed and analyzed with an inverted LSM 510 Zeiss microscope with a 63 X oil immersion lens. Individual cell images (n = 10 for each condition) were acquired with identical settings for laser power, photomultiplier gain, offset, and a fixed pinhole size. The images were analyzed using ImageJ software. A threshold for each channel was selected to avoid background, and the integrated fluorescence intensity was calculated. Total fluorescence was calculated by adding the fluorescence of the permeabilized and non-permeabilized channels.

### Identification of ApoER2-CTF

The ApoER2-expressing cells lines, SNX17 knockdown cells, or controls were lysed in 1% Triton X-100 in PBS containing protease inhibitors, and the proteins in the lysates were resolved in Tris/Tricine gels and analyzed by western blot using an antibody that recognizes the cytoplasmic tail of ApoER2 [24]. To inhibit γ-secretase, the cells were incubated for 16 h with 10 μM DAPT or DMSO as a control. For neurons, 1.5×10^6^ mouse dissociated cortical neurons were infected with lentivirus expressing shRNA for SNX17 or control (pLKO) at DIV 4 with a MOI (multiplicity of infection) of 1. Three days after infection, the cells were lysed as described above.

### Determination of the γ-secretase activity

The γ-secretase activity was assayed *in vitro* using an APP-CTF-derived intramolecularly quenched fluorescent peptide (Calbiochem) according to the manufacturer's instructions and as described [44]. Briefly, cellular membranes from N2a cells (SNX17 knockdown and control, expressing HA-ApoER2) were solubilized in CHAPSO buffer (50 mM Tris–HCl, 2 mM EDTA, protease inhibitors, 0.25% CHAPSO, pH 6.8), followed by incubation at 37°C for different times in 150 μL of assay buffer (50 mM Tris–HCl, protease inhibitors, 2 mM EDTA, 0.25% CHAPSO, pH 6.8, and 8 μM fluorescent APP-CTF-derived peptide). After incubation, the reaction mixture was centrifuged (16,100×g, 15 min) and the supernatant transferred to a 96-well plate. Fluorescence was measured using a PerkinElmer Luminescence spectrometer LS50B (excitation/emission at 350/440 nm). The specific γ-secretase activity was determined after subtracting the fluorescence obtained in the presence of DAPT (10 μM). Background fluorescence was calculated by separately incubating 50 μg of CHAPSO-solubilized P2 membranes and 8 μM APP-CTF-derived peptide with assay buffer for different times and mixing them just before fluorescence determination.

### Production of reelin

Recombinant mouse reelin was obtained from HEK 293 cells stably expressing the full-length protein. Cells were cultured to produce reelin-conditioned medium exactly as described [6], [13]. Mock conditioned medium was prepared using the same protocol from control HEK 293 cells. Briefly, cells were cultured until 80% confluent in high glucose DMEM with 10% FBS containing penicillin and streptomycin and 0.5 mg/mL of G418 at 37°C. After washing two times with PBS, the cells were cultured in high glucose DMEM for an additional 24 h. The cell medium was collected and centrifuged at 1000 rpm for 5 min, and the supernatant was stored at 4°C. This procedure was repeated two more times. The collected medium was concentrated using Amicon ultra-15 centrifugal filter units (filter membrane, 100 kDa).

### ApoER2 degradation

The effects of SNX17 silencing on the ApoER2 half-life under non-stimulated conditions were determined by the detection of mature and immature ApoER2 by western blot of cell lysates from silenced and control N2a cells and by pulse-chase experiments. These experiments were performed in HEK 293 clones transfected with HA-ApoER2 and RAP. Cells were trypsinized and plated in 6-well plates (5×10^5^ cells per well). Two days after transfection, the cells were washed with depletion medium and incubated with 150 μCi of [^35^S]Met/Cys per well for 90 min, followed by chasing in medium without [^35^S]Met/Cys and a 10-fold excess concentration of cold Met and Cys for different times. After each time point, cells were lysed in 1% Triton X-100 in PBS and incubated with anti-HA at 4°C for 4 h. The immune complexes were recovered with protein A-agarose beads. Immunoprecipitated proteins were released from the beads by boiling in Laemmli sample buffer under reducing conditions and were analyzed by SDS-PAGE and autoradiography.

To evaluate the degradation of the receptor involved in reelin internalization, N2a cells stably expressing HA-ApoER2 and silenced for SNX17 were treated with 100 μM CHX for 6 h. Cells not treated with CHX were used as a control. The cells were serum-depleted for 1 h and then incubated with conditioned media with reelin or control, with or without CHX, for 5 h. At the end of the treatment, cells were lysed in 1% Triton X-100 in PBS containing protease inhibitors, and the proteins (ApoER2 full-length) were resolved in Tris/Tricine gels and analyzed by western blot.

### Signaling in neurons

For measurement of Dab1 and cofilin phosphorylation in response to reelin, 1×10^5^ mouse dissociated cortical neurons were transfected at DIV 4 with a GFP expression plasmid and the corresponding shRNA plasmid (0.3 μg each) using Lipofectamine 2000. After 3 days, cells were treated with reelin for 20 min at 37°C. The cells were fixed with 4% PFA and 4% sucrose for 20 min and processed for immunofluorescence using anti-βIII-tubulin antibody (1∶2000 dilution) and anti-phospho- Dab1 (1∶250) or anti-phospho- cofilin (1∶500). The cells were then stained with the appropriate secondary antibodies, and images of individual cells (n = 10 for each condition) were captured with an inverted LSM 510 Zeiss microscope with a 63 X oil immersion lens. The images were analyzed using ImageJ software. The thresholds for each channel were selected to avoid background, and the integrated fluorescence intensity of the soma was calculated. All data were analyzed with GraphPad Prism 4 software using Student's t-test, and displayed as the mean +/- SEM.

The activation of different targets of the reelin signaling pathway was determined by western blot, as previously described [52]. Specifically, 1.5×10^6^ mouse dissociated cortical neurons were infected at DIV 4 with a MOI (multiplicity of infection) of 1. Three days after infection, the cells were incubated with concentrated recombinant reelin for 20 min at 37°C and were lysed in 1% Triton X-100 in PBS containing protease and phosphatase inhibitors, okadaic acid, sodium fluoride, β-glycerol phosphate, and sodium orthovanadate. Dab1 was immunoprecipitated by incubating the cell lysate with protein A-sepharose pre-bound to Dab1 antibody for 4 h at 4°C. The beads were washed three times with PBS, boiled in 2X sample buffer, separated by SDS-PAGE, and total and phosphorylated Dab1 were analyzed by western blot. The presence of SNX17, total and phosphorylated AKT and GSK3β were determined in the lysates. The effect of reelin on the dendritic outgrowth was determined in dissociated hippocampal neurons. Briefly, 1×10^5^ mouse neurons were transfected with a GFP expression plasmid and the corresponding shRNA plasmids (0.3 μg each) after attachment at DIV 0, using Lipofectamine 2000. The cells were treated with recombinant reelin from DIV 2 to DIV 4, with the reelin being replenished every 24 h. At DIV 5, cells were fixed with 4% PFA and 4% sucrose for 20 min and processed for immunofluorescence with anti-βIII-tubulin antibody to identify neurons. Individual cell images of βIII-tubulin and GFP-positive cells (n = 20 for each condition) were captured with an LSM 510 Zeiss inverted microscope with a 63 X oil immersion lens. The images were analyzed using ImageJ software; stacks were reconstructed as one image, and the GFP channel images were analyzed with the ImageJ Sholl analysis plugin using a 10-μm radius increase. Number and length quantitation was performed by making tracings using the Neuron J plugin [53].

All data were analyzed with GraphPad Prism 4 software using Student's t-test, and the data are expressed as the mean +/− SE.

## Results

### The carboxy-terminal domain of SNX17 interacts with the NPxY motif of ApoER2

**Figure 1:**
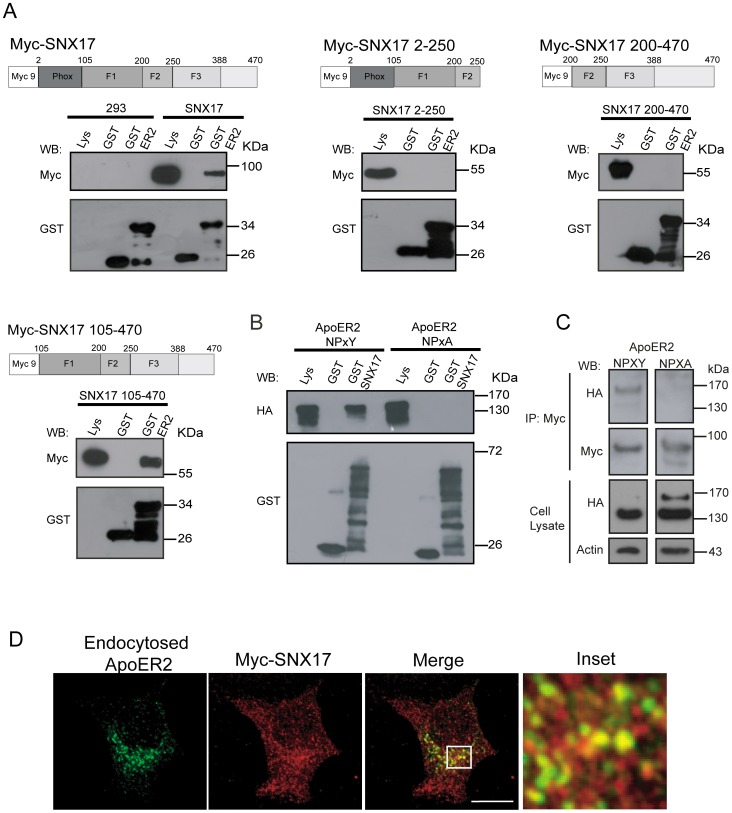
SNX17 interacts with the NPxY motif of ApoER2, and both proteins colocalize after receptor endocytosis. (**A**) HEK 293 cells were transfected with different myc-tagged SNX17 constructs, and their lysates were used for GST pull-down assays using GST or GST-ApoER2. The presence of SNX17 was evaluated by western blot with an anti-myc antibody. GST fusion proteins were detected by western blot using anti-GST antibody. F1, F2 and F3 indicates the three subdomains or modules of the FERM domain [35].(**B**) HEK 293 cells were transfected with HA-ApoER2 wild-type or mutated (NPxY/A) constructs. Cell lysates were used for a pull-down assay using GST or GST-SNX17. The receptor was evaluated by detecting the HA epitope. In both cases, Lys corresponds to 10% of the cell lysate used for the pull-down assay. (C) Cell extracts obtained from cells transiently transfected with myc-SNX17 and HA-ApoER2 were immunoprecipitated with anti-myc and probed for ApoER2 with the anti-HA antibody. Lys corresponds to 2% of the cell lysate used for the coinmunoprecipitation. (**D**) HeLa cells were transfected with HA-ApoER2, RAP, and myc-SNX17. Cells were incubated with anti-HA antibody for 1 h at 4°C, and receptor internalization was allowed for 10 min at 37°C. Cells were fixed and analyzed by immunofluorescence. Bar, 10 μm.

SNX17 is a multidomain adaptor protein containing a PX domain, an atypical FERM domain containing three subdomains or modules (F1. F2, F3), and a carboxy-terminal region [30], [31], [35]. In a similar experiment as that previously performed to investigate how SNX17 binds to LRP1 [31], we performed a series of GST pull-down assays to identify the region of SNX17 that interacts with ApoER2. Recombinant proteins consisting of the full-length cytoplasmic domain of ApoER2 fused to GST or GST alone as a control were incubated with total lysates of HEK 293 cells transfected with different constructs of myc-tagged SNX17 (**Figure 1A**). The interactions were analyzed by western blot using an antibody recognizing the myc epitope. As was previously reported, we observed that full-length SNX17 interacts with ApoER2 [27]; however, the truncated protein carrying the PX and the F1-F2 FERM subdomains (SNX17 2-250) was unable to interact with GST-ApoER2, suggesting that the F3 region of the FERM domain and/or the C-terminal region of the protein are required for the interaction with the receptor. Furthermore, the deletion construct that only lacks the PX domain (SNX17 105–470) but contains the complete FERM domain and C-terminal region of SNX17 interacts with ApoER2. Interestingly, the deletion of the F1 region of the FERM domain (SNX17 200–470) produces a complete loss of ability to interact with ApoER2. Both results indicate that the N-terminal region of FERM domain, (the F1 module), and the C-terminal region of SNX17 (F3 module and C-terminal region) are absolutely essential for the interaction with ApoER2. Similar to other members of the LDL-R family, ApoER2 contains a multifunctional NPxY motif, which is responsible for its signaling [4], [11], endocytosis [24] and also the role of facilitating the interaction with several adaptor proteins [54], [55]. Using full-length SNX17 fused to GST, we determined that the interaction with ApoER2 was dependent on the integrity of the NPxY motif, as SNX17 was not able to interact with the mutated receptor containing the NPxA motif (**Figure 1B**). To determine whether this interaction occurs within the cells, we performed a co-immunoprecipitation of myc-SNX17 with either wild-type or mutated ApoER2-HA in HEK293.When the immunoprecipitates were blotted with anti-HA, we found that only wild type and the mature form of ApoER2 co-immunoprecipitated with SNX17, but not the mutant NPxA or immature (low molecular weight form) receptor (**Figure 1C**).

### SNX17 participates in ApoER2 recycling from a Rab5 early endosomal compartment to a Rab11 recycling compartment but does not regulate constitutive receptor degradation

Because SNX17 has been associated with different steps of the endocytic pathway [30], [31], we asked whether ApoER2 transits across an SNX17-positive compartment during its endocytic trafficking. HeLa cells were co-transfected with HA-ApoER2 and myc-SNX17. Cell surface receptors were labeled with an anti-HA antibody at 4°C and were then allowed to be internalized at 37°C. As shown in **Figure 1D**, the internalized ApoER2 shows a clear colocalization with SNX17 after 10 min of endocytosis.

**Figure 2:**
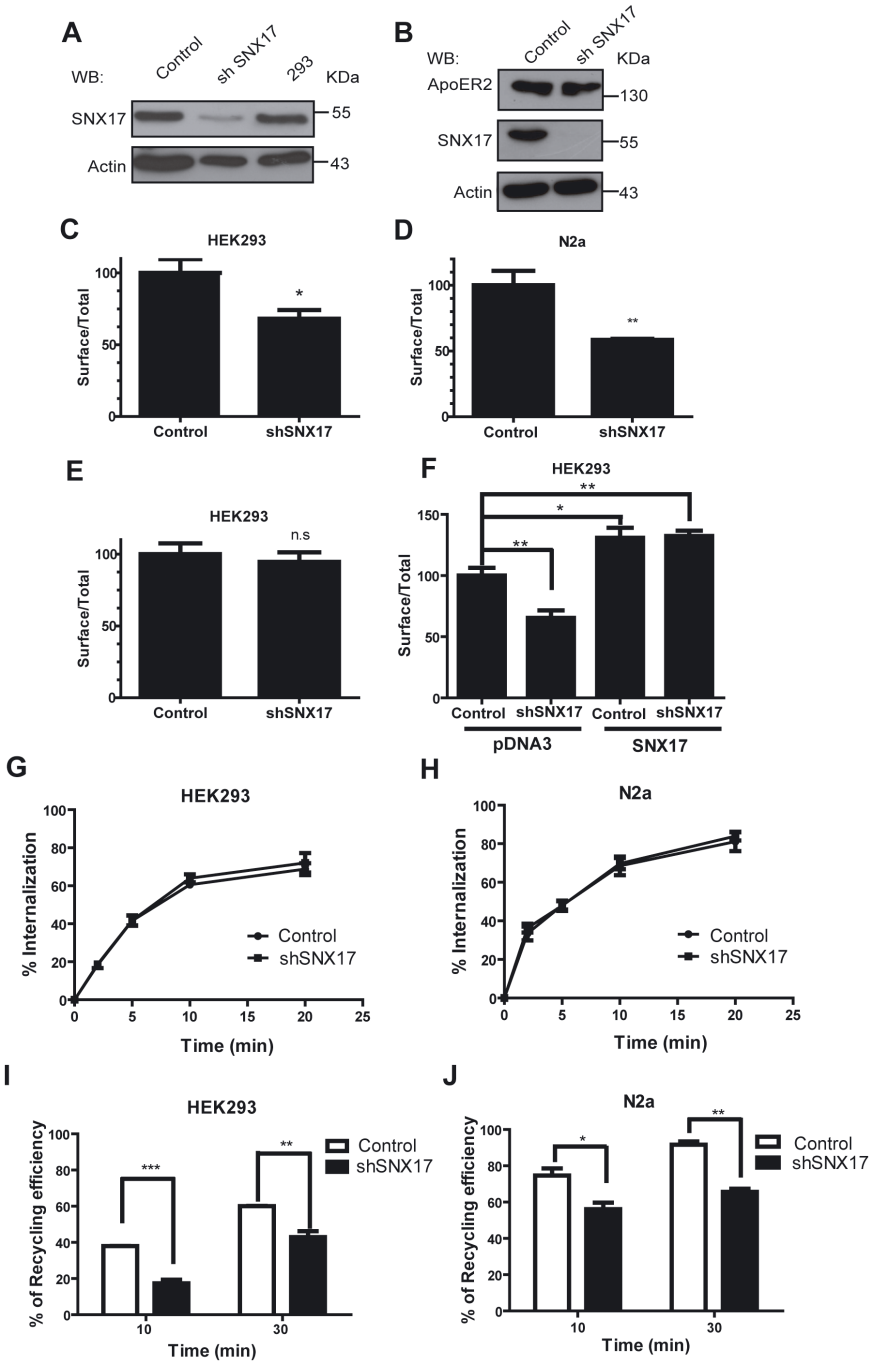
SNX17 knockdown diminishes surface levels of ApoER2 by decreasing its recycling. (**A**) HEK 293 cells or (**B**) N2a cells infected with a lentiviral vectors expressing shRNA against human or mouse SNX17 or empty pLKO vector were transfected with HA-ApoER2. Cells were lysed with 1% Triton X-100 in PBS and analyzed by western blot. (**C**) HEK 293 clones transfected with HA-ApoER2 and RAP were used to analyze the ratio of cell surface to total ApoER2 by FACS, as described in the Methods section. The graphic shows the ratio of the values of non-permeabilized versus permeabilized cells considering the control condition as 100%. (**D**) N2a clones expressing ApoER2 and SNX17 silenced or control were treated as in C. (**E**) Control or SNX17 knockdown HEK293 clones were transfected with a plasmid for mMeg4, a construct of megalin carrying the fourth ligand binding domain, the transmembrane domain, and the cytosolic domain. The receptor was determined in these cells by FACS. (**F**) SNX17 knockdown HEK293 clones expressing HA-ApoER2 were transfected with a shRNA-resistant mouse myc-SNX17. The presence of ApoER2 and SNX17 proteins were analyzed using a chicken anti-HA antibody and a mouse anti-Myc antibody respectively, in permeabilized and non-permeabilized conditions. (**G**) Control or SNX17 knockdown HEK293 clones expressing HA-ApoER2 were incubated with an Alexa 488-conjugated anti-HA antibody for 1 h at 4°C. The temperature was increased to 37°C for the indicated time period, and theremaining surface bound antibody was removed with an acid wash. The intracellular antibody was detected in the cells by flow cytometry. The total antibody bound to the cell was also determined. The endocytic rate was calculated by subtracting the value of the cells exposed to the acid wash at time 0 (A_0_) from each time point, and dividing by A_0._ (**H**) The same experimental procedure described in G was performed with control or SNX17 knockdown N2a cells expressing HA-ApoER2. (**I**) The same HEK293T cells used in G were utilized in a recycling experiment. The cells were labeled with an Alexa 488-conjugated anti-HA antibody for 30 min at 37°C. The recycled receptor was then chased at the surface with a quenching anti-Alexa 488 antibody. The cells were analyzed by flow cytometry, and the percentage of the initial fluorescence remaining at each time point was calculated as the difference in the fluorescence between time 0 and each chased time point. Every time (including time 0) was normalized to the non-chased value. The percentage of recycling efficiency was calculated by subtracting the percentage of internal fluorescence from 100. (**J**) Control or SNX17 knockdown N2a clones expressing HA-ApoER2 were treated as in C. *p<0.05, **p<0.01, ***p<0.001.

To address whether SNX17 has an active role in the endocytic behavior of ApoER2, we silenced SNX17 in HEK 293 cells using a lentivirus system expressing shRNA against human SNX17 and also generated a control cell line by infecting the cells with a control lentivirus (**Figure 2A**). To identify the global effect of SNX17 knockdown on ApoER2 trafficking, we compared the surface levels of the receptor in silenced and non-silenced cells. The silenced and non-silenced HEK293 cells were transfected with the HA-ApoER2 expression plasmid, and the surface and intracellular levels of the receptor were determined by FACS analysis. As shown in **Figure 2C**, silencing of SNX17 diminished the ApoER2 surface level by 30%, indicating a possible role of the adaptor protein in ApoER2 trafficking. We also analyzed the effect of SNX17 knockdown in a neuronal cell type, the neuroblastoma cell line N2a. These cells were stable transfectants expressing human ApoER2 that were infected with a mouse shSNX17 or pLKO control lentivirus (**Figure 2B**). As shown in **Figure 2D**, knockdown of SNX17 induced a significant (almost 50%) decrease in the surface level of ApoER2. To determine that the reduction of cell surface ApoER2 was not a result of a global effect of knockdown on the endocytic pathway, we evaluated the trafficking behavior of megalin/LRP2 that is unable to interact with SNX17 [27], [37]. HEK 293 cells, silenced or control for SNX17, were transfected with a construct of mini-megalin, mMeg4, which is composed of the fourth ligand binding domain, the transmembrane domain, and the cytosolic domain of the receptor [56]. The cell surface levels of mini-megalin were not modified upon SNX17 knockdown (**Figure 2E**), indicating that the previously observed effect was specific to ApoER2. To confirm that the decrease in ApoER2 at the cell surface was produced by the deficiency in SNX17, we measured the cell surface levels of ApoER2 in SNX17-silenced HEK 293 cells that were transfected with a construct of myc-SNX17 from mouse. The presence of myc-SNX17 not only restored the surface levels of ApoER2 in silenced cells but also induced an increase in the surface levels of ApoER2 (**Figure 2F**). The functional recovery was correlated with the expression levels of SNX17 (**Figure S1 A**). This effect was dependent on the simultaneous presence of the PX domain and FERM domains of SNX17, because neither the expression of the SNX17 truncated form 2–250 that lacks FERM F3 subdomain, nor the 105–470 that lacks the PX domain but has the complete FERM domain, were able to recover the cell surface levels of ApoER2 (**Figure S1 B,C**).

A reduction in the cell surface levels of ApoER2 could be explained by an increase in the endocytosis rate of the receptor considering the data published indicating that SNX17 regulates P-selectin endocytosis [30]. Therefore, we analyzed the effect of SNX17 downregulation on ApoER2 endocytosis in the SNX17-silenced cells compared to the control (non-silenced) cells. The internalization kinetics of the receptor was monitored by following the internalized anti-HA antibody labeled with Alexa 488 at different times by FACS. As shown in **Figure 2G**, the receptor was internalized with the same kinetics in the presence and absence of SNX17 in HEK293 cells, indicating that this adaptor protein does not participate in the initial endocytosis of ApoER2. Similar results were obtained in SNX17 knockdown N2a cells (**Figure 2H**).

SNX17 does not regulate ApoER2 internalization, therefore we deducted its function in the recycling of ApoER2, given that other previous reports relate SNX17 to LRP1 recycling [31], [36]. ApoER2 recycling was determined as previously described [31], [36], [56]. In SNX17 knockdown cells, there was a significant (almost 30%) decrease in ApoER2 recycling (**Figure 2I**). This result is in agreement with the previous observation of a reduction in ApoER2 cell surface levels (**Figure 2C**). Although in N2a cells the receptor recycles more efficiently than in HEK 293 cells, the silencing of SNX17 induced a similar decrease in ApoER2 recycling (**Figure 2J**). These results indicate a role for SNX17 in the endocytic recycling of ApoER2.

**Figure 3:**
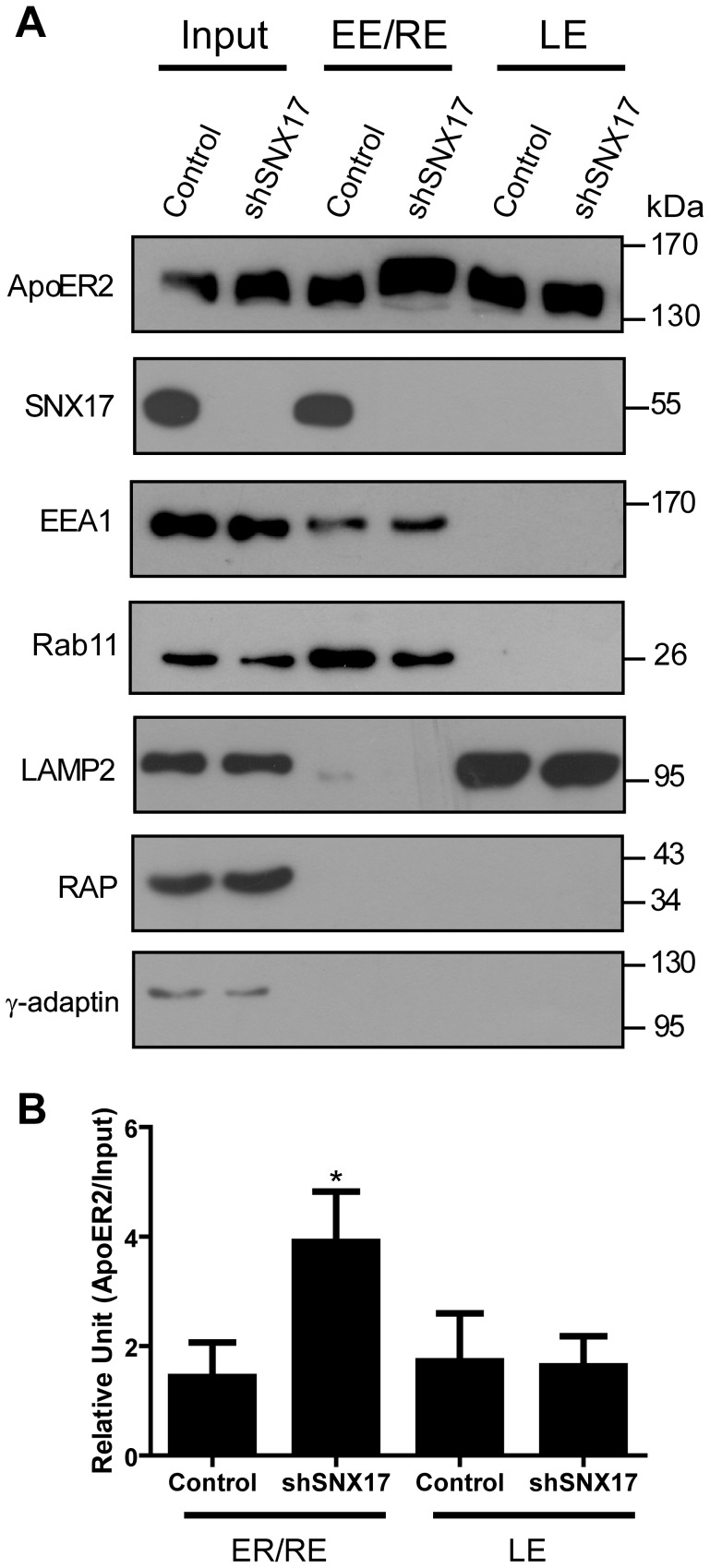
SNX17 knockdown induces the retention of ApoER2 in early/recycling endosomes. (**A**) Control (pLKO) or SNX17 knockdown N2a cells expressing ApoER2 were subjected to subcellular fractionation to isolate endosomal compartments by a discontinuous sucrose gradient. Samples of the different fractions were resolved in Tris/Tricine gels and analyzed by western blot. The input corresponds to 5% of the lysate. EE/RE: early endosome/recycling endosome; LE: late endosome; fractions were free of endoplasmic reticulum (RAP) or Golgi (γ-Adaptin) contamination. (**B**) Quantification of ApoER2 in different compartments normalized by the Input. *p<0.05.

Because there is more intracellular ApoER2 when the level of SNX17 is reduced, we decided to identify the compartment in which ApoER2 is retained in the SNX17 knockdown cells. Control or shSNX17 N2a cells expressing HA-ApoER2 were used for subcellular fractionation. When SNX17 was silenced, there was a significant increase in ApoER2 in the fractions corresponding to early/recycling endosomes (**Figure 3A,B**). In agreement with previous reports, SNX17 was present in the fraction containing early endosomal membranes. [27]. The subcellular endocytic fractions in which both SNX17 and ApoER2 were present were negative for the Golgi marker γ-adaptin and the endoplasmic reticulum marker RAP.

**Figure 4:**
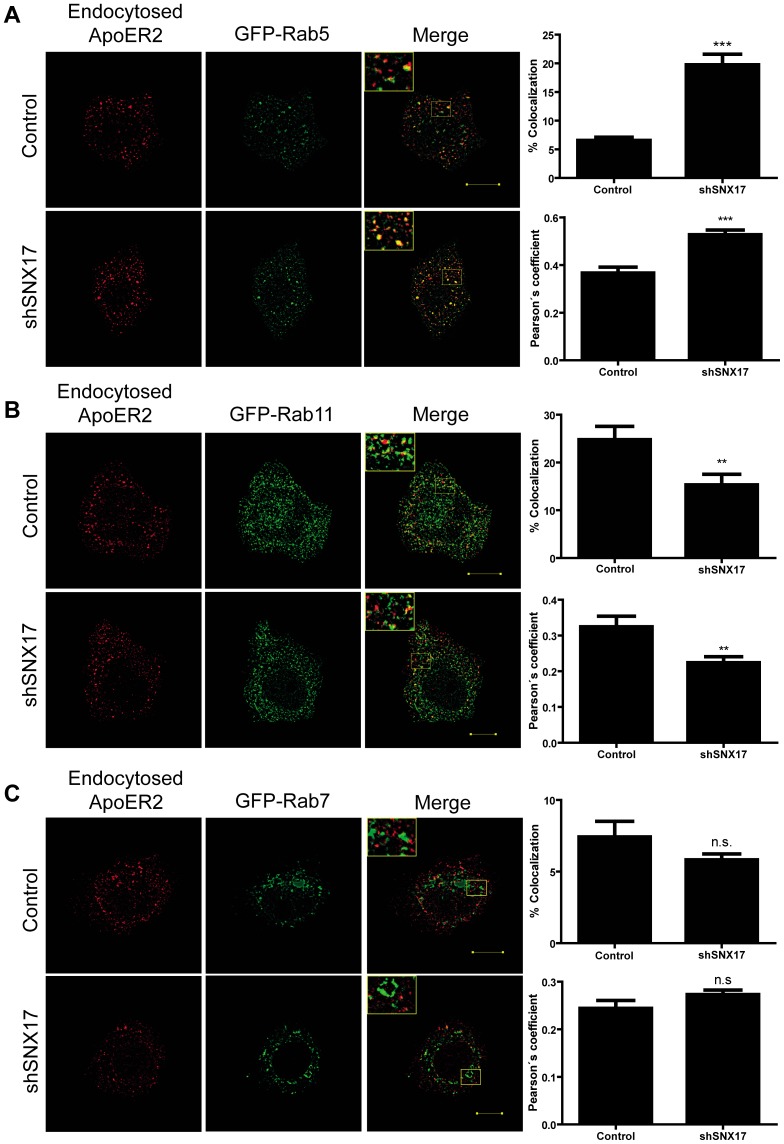
SNX17 facilitates ApoER2 trafficking from the early endosome to the recycling endosome. HeLa cells silenced for SNX17 or control pLKO cells were transfected with HA-ApoER2, RAP, and GFP-Rab5 (**A**); GFP-Rab11 (**B**); or GFP-Rab7 (**C**). Two days after transfection, cells were incubated with anti-HA antibody for 1 h at 4°C, incubated for 40 min at 37°C to allow for receptor internalization, and the remaining surface receptor was removed by acid wash. Cells were washed, permeabilized, and incubated with Alexa 594-conjugated goat anti-mouse IgG. Images were captured by confocal microscopy, and Mander's colocalization index and Pearson's coefficient were calculated in 10 cells for each condition. **p<0.01, ***p<0.001. Bars, 10 μm.

**Figure 5:**
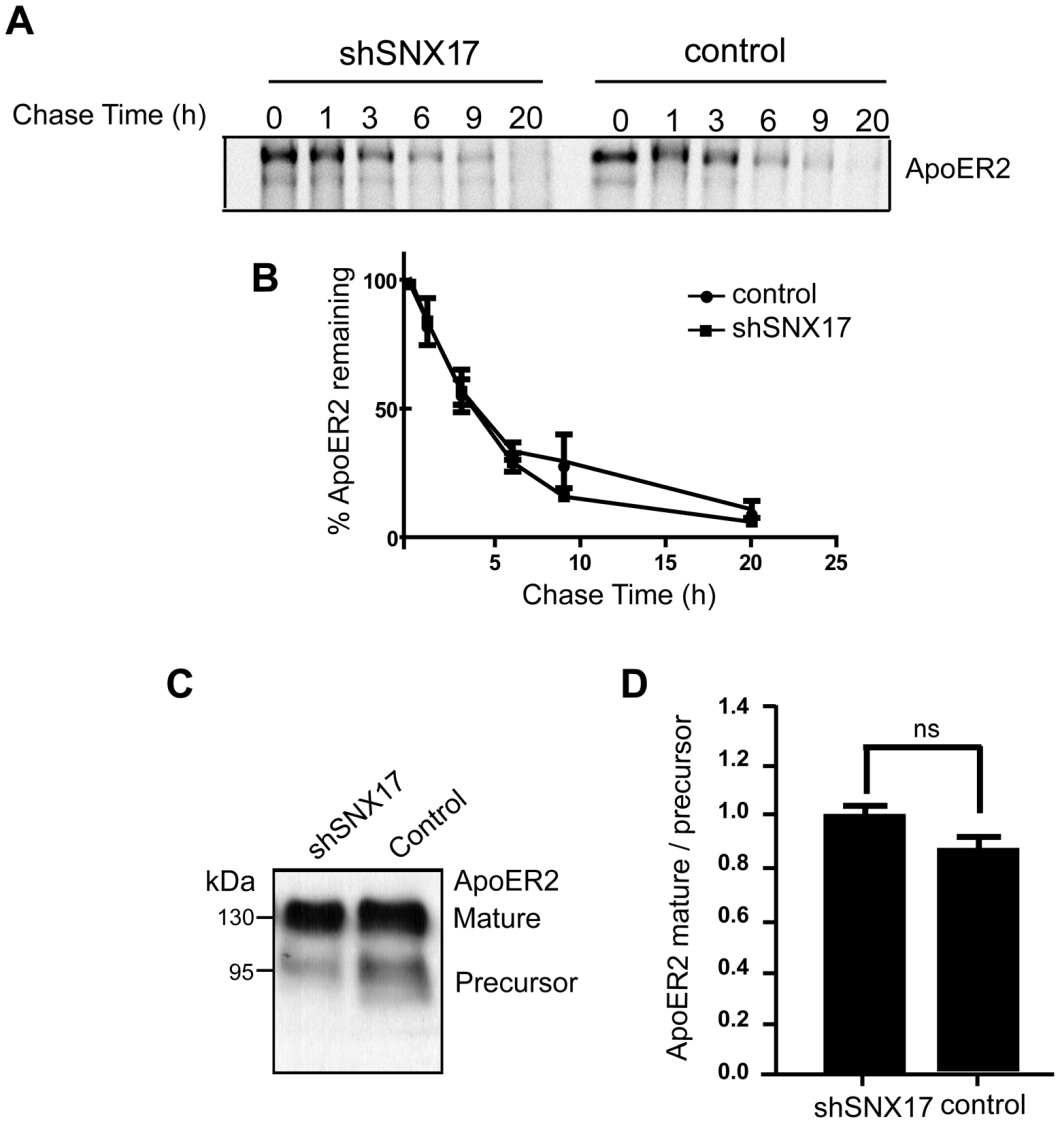
SNX17 does not regulate the half-life of ApoER2 under basal conditions. (**A**) HEK 293 cells treated with shRNA against SNX17 and pLKO control clones were transfected with HA-ApoER2 and RAP, incubated with medium containing [35S]Met/Cys for 90 min, and chased with medium without [35S]Met/Cys for 1, 3, 6, 9, and 20 h, followed by immunoprecipitation with anti-HA antibody. The samples were separated by SDS-PAGE and subjected to autoradiography. (**B**) The radioactive intensity was plotted against time. In both conditions, ApoER2 is degraded with similar kinetics. (**C**) Western blot of equal amounts of cell lysate of N2a cells (pLKO and SNX17 KD) expressing ApoER2, which was detected with anti-HA antibody. The ER precursor and the mature fully glycosylated forms are shown. (**D**) There is no difference in the amount of mature form with respect to the precursor in SNX17 knockdown cells indicating that ApoER2 degradation was not affected by the lack of SNX17 (n = 3).

The subcellular fractionation protocol does not allow us to discriminate if ApoER2 is retained in an early or recycling endosomal compartment. Therefore, we performed colocalization analysis of the endocytosed receptors with Rab5 (early endosome marker) and Rab11 (recycling endosome marker). SNX17 knockdown HeLa cells were transfected with HA-ApoER2 and GFP-Rab5, GFP-Rab11 or GFP-Rab7 (to identify late endosomes). ApoER2 was labeled at the cell surface with anti-HA antibody for 2 h at 4°C, followed by incubation of the cells for 40 min at 37°C to allow for its internalization and endosomal distribution. The antibodies remaining at the cell surface were removed by an acid wash. The cells were fixed and analyzed by confocal microscopy to determine the percentage of receptor colocalization with the different Rabs. As shown in **Figure 4A**, reduced levels of SNX17 resulted in endocytosed ApoER2 that colocalized significantly more often with Rab5 and also showed a significant decrease in colocalization with Rab11 compared to the control condition (**Figure 4B**). These results implicate a role for SNX17 in the endocytic trafficking of ApoER2 from early endosomes to recycling endosomes. It is noteworthy that the colocalization of ApoER2 and Rab5 (approximately 68%) was not altered between the silenced and control cells after 10 min of receptor endocytosis (**Figure S2**), again indicating that SNX17 does not participate in the internalization rate or the arrival of the receptor to the early endosome compartment. In agreement with the subcellular fractionation results, we did not observe a major difference in the colocalization of ApoER2 with the late endosome marker GFP-Rab7 in the silenced cells compared with the control cells (**Figure 4C**). Furthermore there was no change in the half-life of ApoER2 determined by a pulse-chase protocol followed by immunoprecipitation (**Figure 5A,5B**). In addition, the ratio of precursor receptor present in the endoplasmic reticulum (ER) to the mature form was similar in both the silenced and control cells, indicating that SNX17 does not regulate the constitutive degradation of ApoER2 (**Figure 5C,5D**).

### Reduction in the level of SNX17 is associated with an increased level of the ApoER2 carboxy-terminal fragment

**Figure 6:**
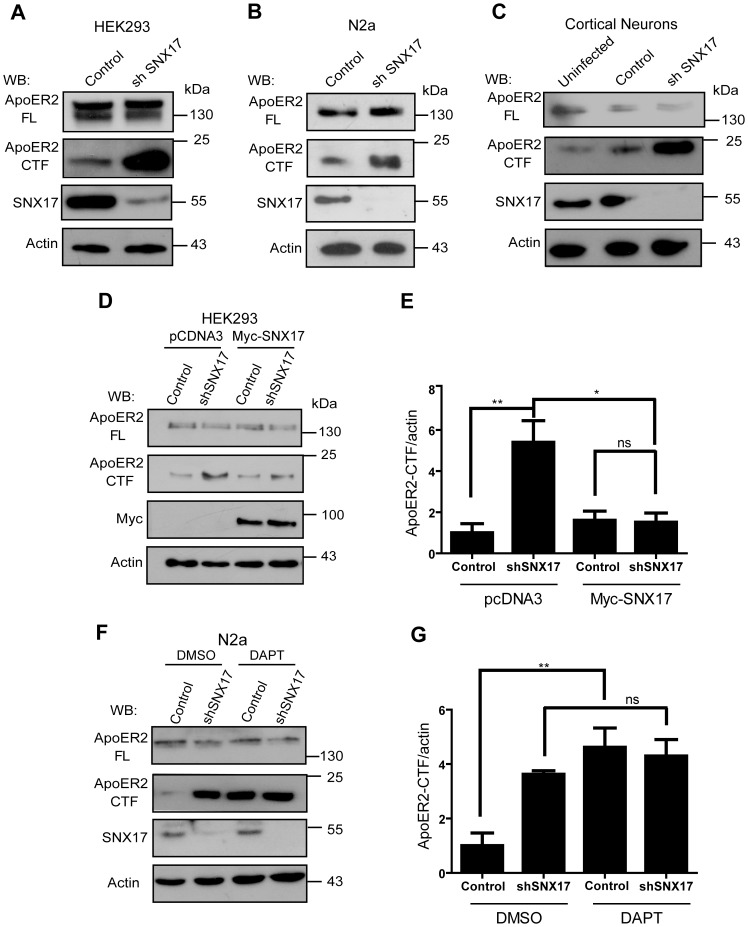
SNX17 knockdown increases the ApoER2 CTF level. Control or SNX17 knockdown (**A**) HEK293 cells and (**B**) N2a cells expressing HA-ApoER2 were lysed in PBS-T. The samples were separated on Tris-Tricine gels and analyzed by western blot. (**C**) DIV 4 cortical mouse neurons were infected with a lentiviral vector expressing SNX17 shRNA or empty pLKO at an MOI of 1. Three days after the infection, the cells were lysed and the lysates were resolved on Tris-Tricine gels. (**D**) Control or SNX17 knockdown HEK293 cell clones were transfected with the ApoER2 plasmid and either the mouse myc-SNX17 plasmid or pcDNA3 as a control. The resultant cell lysates were analyzed by western blot. (**E**) The band intensity was quantified to determine the ApoER2 CTF level normalized to actin. In all conditions, the bands were normalized to the control condition (empty pLKO and pcDNA3). The figure shows the average of three independent experiments. *p<0.05, **p<0.01, ns p>0.05. (**F**) Control or SNX17 knockdown N2a cells expressing ApoER2 were treated with DAPT or DMSO for 16 h, and then the presence of ApoER2 and its CTF were determined by western blot. (**G**) Quantification was performed as previously described, using pLKO cells treated with DMSO as the control condition. **p<0.001 and ns p>0.05.

Several lines of evidence relate ApoER2 trafficking with its susceptibility to proteolytic processing by the α-secretases of the ADAM family and by the γ-secretase complex [39], [42], [55], [57]. When over expressing adaptor proteins, which are associated with higher levels of receptor at the cell surface, the ApoER2 CTF (the product of sheddases and substrate of the γ-secretase complex) is also detected at a higher level [40], [54]. In our results, we found that the reduction of SNX17 was associated with a lower cell surface level of ApoER2 (**Figure 2C,2D**) and no change in its half-life or total levels were observed (**Figure 5**). Interestingly, when ApoER2 is retained in an early endosomal compartment, we found an increase in the CTF levels of the transfected receptor in two cell lines (HEK 293 and N2a) (**Figure 6A,6B**). Moreover, when the processing of endogenous ApoER2 in primary cortical neurons was analyzed, it was found that the CTF level was also increased in SNX17 knockdown conditions (**Figure 6C**). To confirm that the change in the level of the CTFs was due to a reduction of SNX17, we overexpressed mouse myc-SNX17 in HEK 293 cells that were previously silenced for endogenous SNX17. Under these conditions, ApoER2 processing that was determined by the CTF level, was the same as in the control condition (**Figure 6D,6E**). The level of expression of transfected myc-SNX17 in the knockdown cells was similar to the level of the endogenous adaptor protein in control, non-silenced cells (**Figure S1 D**).

The accumulation of the ApoER2 CTF in SNX17 knockdown cells suggests at least two different scenarios. The first is an increase in the first proteolytic step by α-secretase (shedding), and the second is a decrease in the second proteolytic step by the γ-secretase complex. To gain insight into the underlying mechanism of ApoER2 CTF accumulation upon SNX17 knockdown, we incubated SNX17 knockdown and control N2a cells with DAPT, a γ-secretase inhibitor, and analyzed the ApoER2 CTF level by western blot. As shown (**Figure 6F,6G**), after incubation with DAPT, the CTF level was increased in the control cells as expected. However, the same treatment did not affect/increase the CTF level in the knockdown cells. These results indicate that in cells with reduced levels of SNX17, ApoER2 CTF levels are increased, potentially due to less efficient processing by the γ-secretase complex. Because the total activity of the γ-secretase complex was not diminished in the SNX17 knockdown cells (**Figure S3**), this result suggests that the lack of CTF processing could be due to altered localizations of the substrate (accumulated in early endosome) respect of the enzymatic complex.

### SNX17 regulates reelin-induced ApoER2 degradation

**Figure 7:**
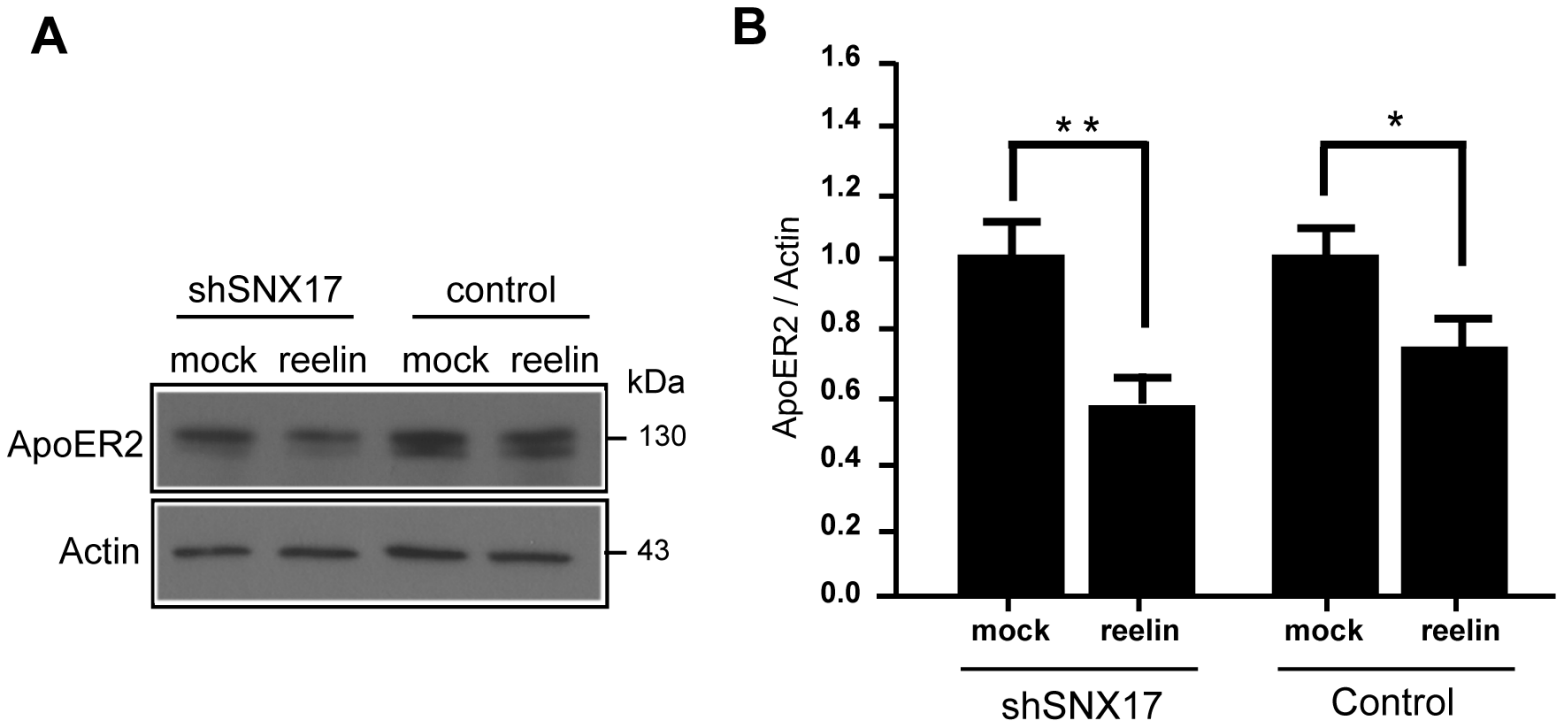
SNX17 regulates the degradation of ApoER2 induced by reelin. (**A**) N2a-ApoER2 cells silenced or not for SNX17 were incubated with 100 μM cycloheximide for 6 h. After 1 h of serum depletion, cells were incubated with the respective conditioned media for 5 h. ApoER2 was detected by western blot. (**B**) Band intensities were quantified to determine the ratio of ApoER2 versus actin as the loading control. The graphic indicates the average of three independent experiments. *p<0.05; **p<0.01.

To verify the role of SNX17 under conditions in which ApoER2 is degraded in the lysosome [43], we compared the total levels of the receptor (precursor and mature forms) or the mature receptor after 5 h of reelin incubation in control versus SNX17 silenced cells. The mature receptor is present predominantly in the endocytic pathway (in particular, the plasma membrane, endosomes, and lysosomes) therefore the cells were pretreated with cycloheximide (CHX) for 1 h before and during incubation with reelin to ensure better detection the mature receptor as normally a significant part of the total receptor is present in the biosynthetic pathway.(**Figure 7A,7B**). The reduced expression of SNX17 was associated with an increase in the reelin-induced degradation of ApoER2, indicating that in normal conditions, SNX17 counteracts the receptor degradation pathway activated by reelin internalization.

### ApoER2 trafficking and function in primary neuronal cultures is altered in the absence of SNX17

**Figure 8:**
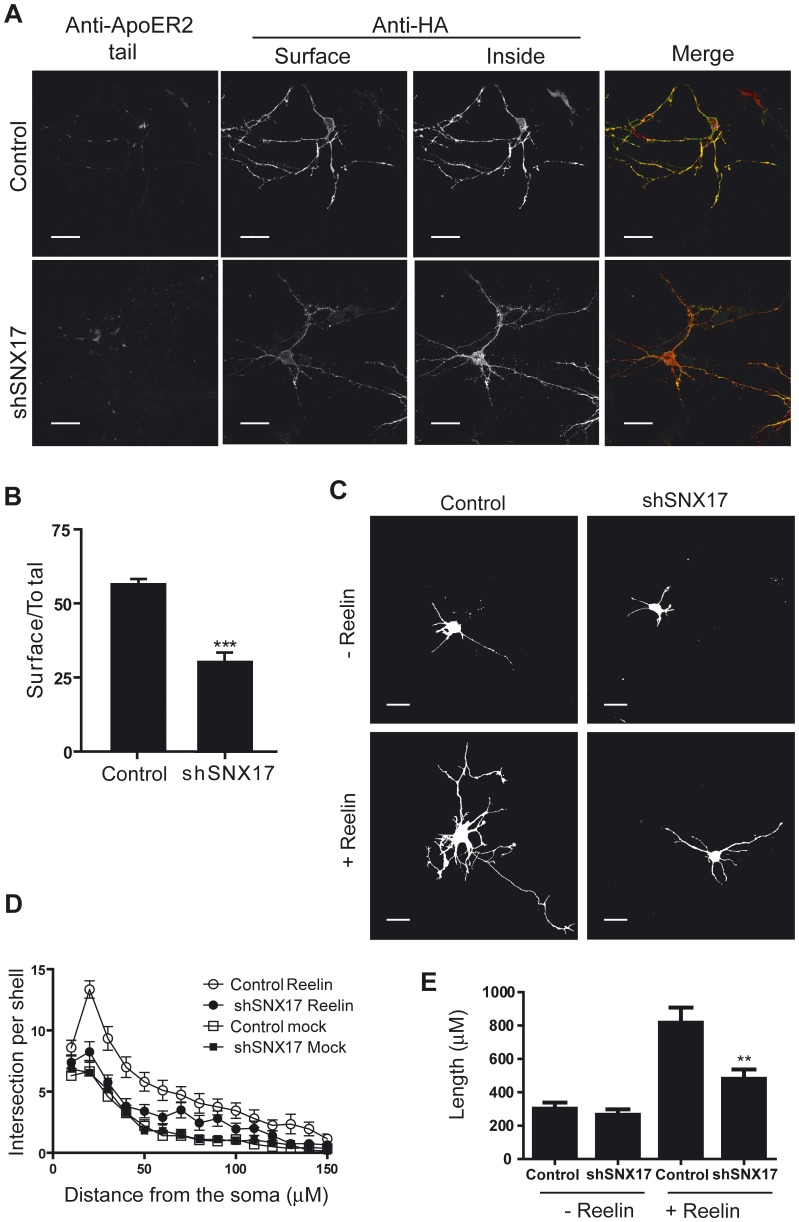
SNX17 knockdown diminishes the surface level of ApoER2 and reelin-induced dendritic development in neurons. (**A, B**) The cell surface level of ApoER2 was determined in DIV 5 mouse cortical neurons co-transfected with HA-ApoER2 and either SNX17 shRNA or pLKO. The cell surface receptor was labeled 48 h after transfection with a mouse anti-HA antibody. To control for the absence of permeabilization, cells were simultaneously incubated with an antibody against the cytoplasmic tail of ApoER2. The intracellular ApoER2 was detected thereafter in the fixed and permeabilized neurons with a chicken anti-HA antibody. Images of individual cells (n = 10 for each condition) were captured by confocal microscopy and analyzed using ImageJ software, selecting the threshold for each channel to avoid background. Total fluorescence was calculated by adding the fluorescence of the permeabilized and non-permeabilized channels. (**C, D**) Mouse dissociated hippocampal neurons were transfected with GFP and the corresponding shRNA, treated with reelin for 3 days, fixed and analyzed by immunofluorescence. Images were captured by confocal microscopy and used for Sholl analysis (n = 20 cells per condition). (**E**) The length of dendrites of reelin treated cells was significantly reduced in SNX17 knockdown neurons *p<0.05; **p<0.01; ***p<0.001. Bars, 20 μm.

Our previous observations in cells lines and the known neuronal role of ApoER2 led us to analyze the effect of SNX17 knockdown in the trafficking and function of ApoER2 in primary cultured neurons. Initially, we tested the ability of the available shRNA to knock down SNX17 and visualize this reduction by indirect inmunofluorescence. Mouse cortical neurons at DIV 5 were cotransfected with a plasmid encoding GFP and either a plasmid containing shRNA against SNX17 or pLKO as a control. Two days later, the neurons were fixed, and SNX17 expression was determined (see **Methods S1**). The neurons transfected with SNX17 shRNA showed a visible reduction in SNX17 expression (**Figure S4**). To analyze the effect of SNX17 on ApoER2 cell surface levels, cortical neurons were co-transfected with plasmids for HA-ApoER2 and SNX17 shRNA. The intracellular and cell surface levels of ApoER2 were evaluated with different anti-HA antibodies (mouse and chicken). As is shown in **Figure 8A,8B**, SNX17 knockdown was associated with a 50% reduction in the cell surface levels of ApoER2, similar to the previous result obtained with the cell lines transfected with the receptor (**Figure 2**).

The neuronal function of ApoER2 was previously shown to be associated with the signaling pathway triggered upon reelin binding. Therefore, we tested whether a reduction in the level of the receptor at the cell surface, which was observed under the SNX17 knockdown condition, affects this signaling pathway. Long-term treatment with reelin stimulates dendritic development in a lipoprotein receptor-dependent and adaptor protein Dab1 phosphorylation-dependent manner [20]. Thus, we analyzed the effect of reduced SNX17 on the dendritic arborization. Neurons were cotransfected at DIV 0 with pLKO containing the shRNA against SNX17 or empty pLKO as a control with an expression plasmid for GFP, and the cells were incubated with reelin from DIV 2 to DIV 4. At DIV 5, dendritic outgrowth was examined using Sholl analysis and dendritic length determination. In SNX17 silenced neurons, both the length and the arborization of the dendrites were significantly reduced (**Figure 8C,8D**). To clarify if this effect is produced by reducing the length and/or the number of dendrites, both parameters were quantified in primary and secondary dendrites (**Figure S5**). In SNX17 knockdown neurons, the number of primary dendrites was not changed, but a reduced number of secondary dendrites were observed under basal and reelin stimulation conditions while shorter primary and secondary dendrites were observed when the neurons were stimulated with reelin.

**Figure 9:**
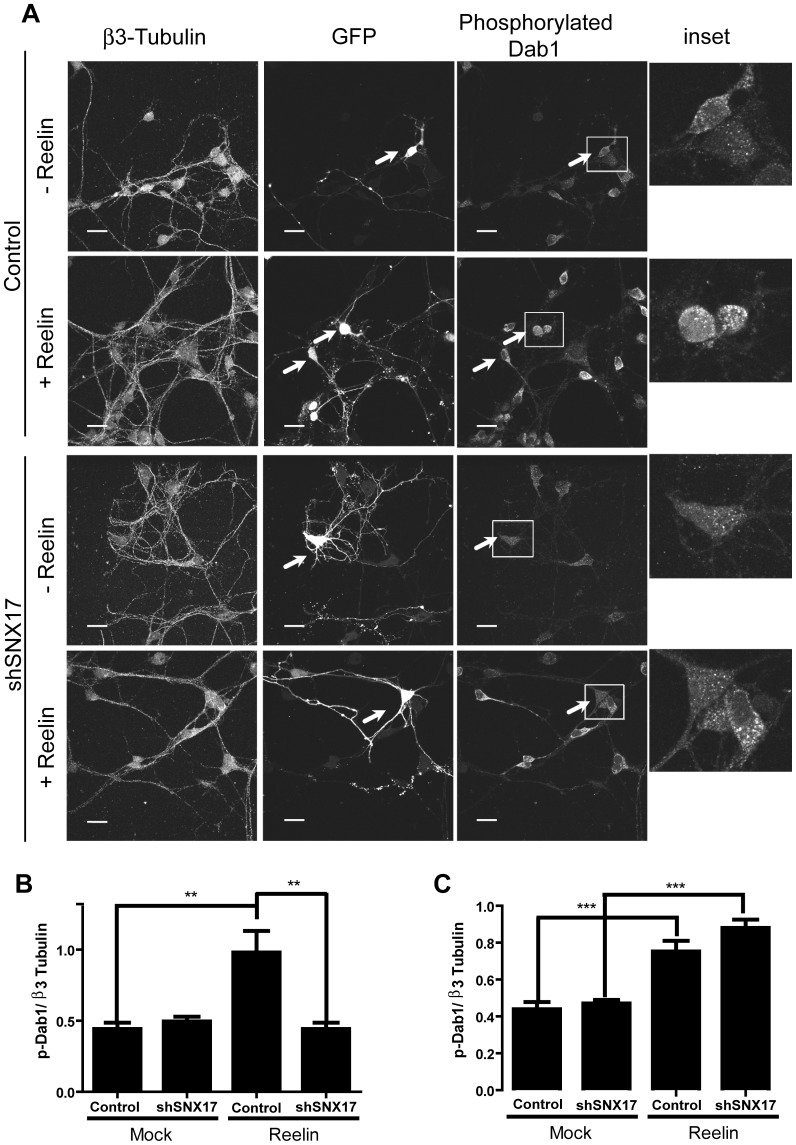
SNX17 has a positive role in the reelin signaling pathway. (**A**) Mouse dissociated cortical neurons were transfected at DIV 4 with a GFP expression plasmid and the corresponding shRNA plasmid. At DIV 7, cells were treated with reelin, fixed, and analyzed by immunofluorescence using anti-phospho-Dab1 and anti-βIII-tubulin antibodies. Images of individual cells were captured, and the integrated fluorescence intensity of the soma was calculated using ImageJ software. Phosphorylation of Dab1 was quantified in cells under each condition, and the intensity of βIII-tubulin was used for normalization. In (**B**), the quantification of the fluorescence intensity is shown, of both cells transfected with pLKO plasmid (control), and with shSNX17 in the presence of reelin (Reelin) or DMSO (Mock). In (**C**), the same quantitative analysis of non-transfected cells present at each experimental condition is shown. **p<0.01, ***p<0.001.

**Figure 10:**
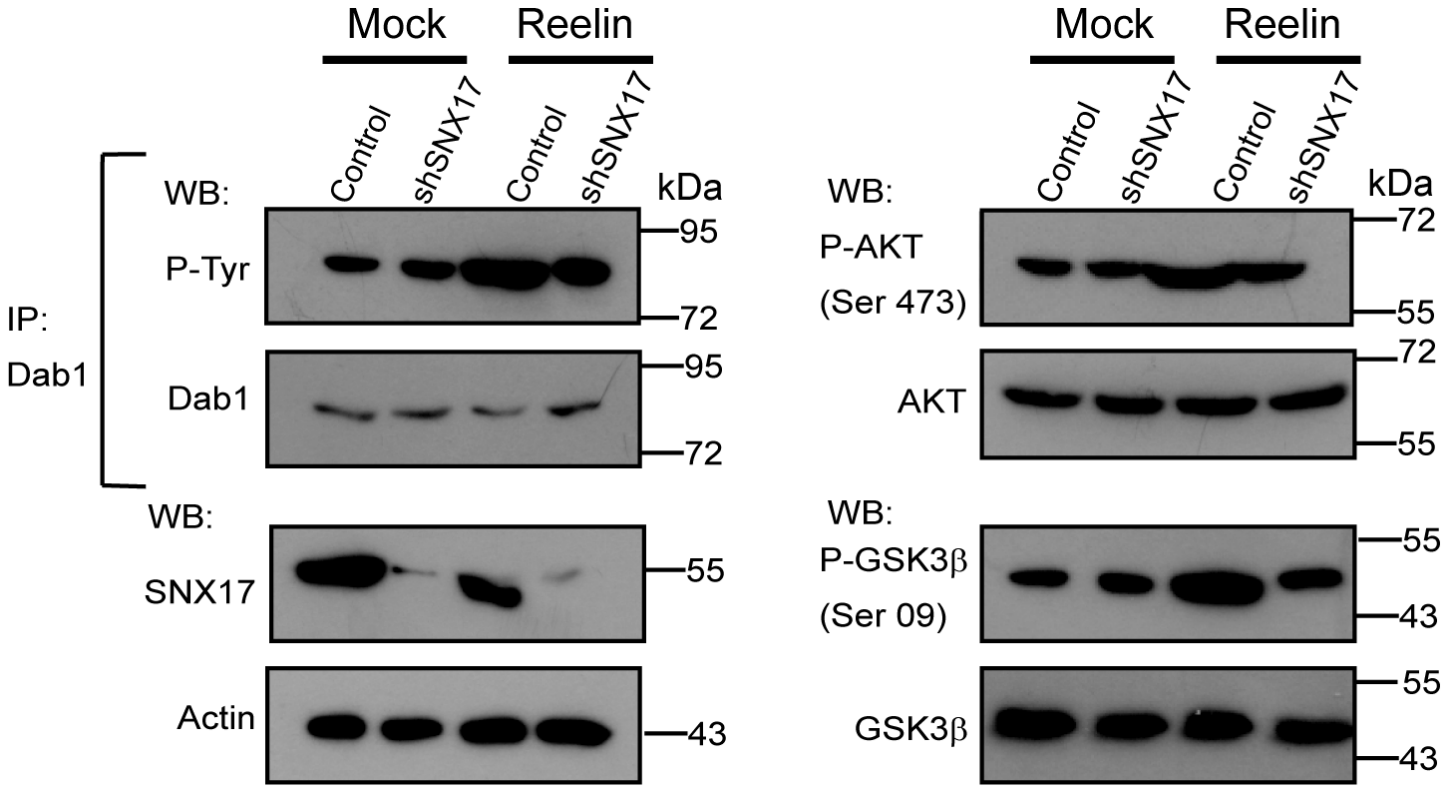
The reelin signaling pathway is impaired in SNX17 knockdown cells. DIV 4 cortical mouse neurons were infected with the a lentiviral system expressing the parental plasmid pLKO (Control) or SNX17 shRNA (shSNX17) at MOI 1; three days after infection, the cells were incubated with reelin-containing medium. Next, cells were lysed and analyzed by western blot. Dab1 was immunoprecipitated (IP) with an anti-Dab1 antibody and analyzed by western blot using an anti-phosphotyrosine antibody. The Dab1 downstream targets AKT and GSK3β were also analyzed by western blot. For all of the proteins analyzed, the reelin-induced phosphorylation was reduced in the SNX17 knockdown cells.

**Figure 11:**
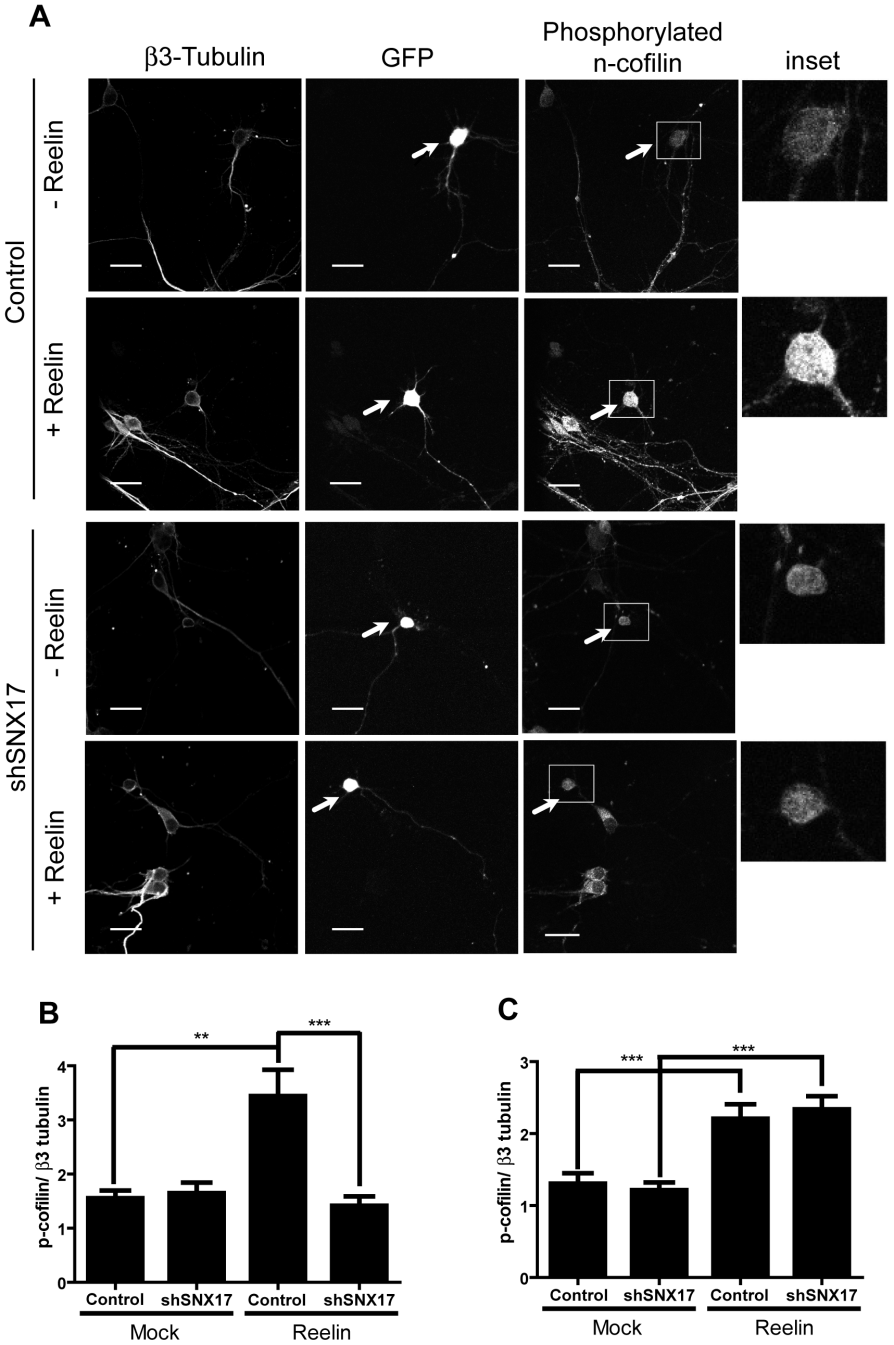
SNX17 knockdown decreases n-cofilin phosphorylation induced by reelin. (**A**) Dissociated mouse cortical neurons were transfected at DIV 5 with a GFP expression plasmid and the corresponding shRNA plasmid. At DIV 7, cells were treated with reelin, fixed, and analyzed by immunofluorescence to detect phospho-cofilin and βIII-tubulin. Images were captured, and the integrated fluorescence intensity of the soma was calculated using ImageJ software. Phosphorylation of n-cofilin was quantified in cells at each condition, and the intensity of βIII-tubulin was used for normalization. In (**B**), the quantification of the fluorescence intensity is shown of both cells transfected with pLKO plasmid (Control), and with shSNX17 in the presence of reelin (Reelin) or with DMSO (Mock). In (**C**), the same quantitative analysis of non-transfected cells present in each experimental condition is shown. **p<0.01; ***p<0.001.

Moreover, we analyzed the requirement of SNX17 for the reelin signaling pathway after a short-term stimulation of cortical neurons. Silenced and control cortical neurons were incubated with conditioned medium containing reelin for 20 min. Knockdown neurons exhibited a considerable decrease in the activation of downstream reelin effectors, specifically the phosphorylation levels of the adaptor protein Dab1 as analyzed by immunofluorescence (**Figure 9**) and by western blot, along with the phosphorylation of AKT and GSK3β (**Figure 10**). Considering that reelin induces Dab1 phosphorylation through the activation of VLDLR, even though ApoER2 is the predominant receptor in these neurons, we tested the activation of ApoER2 induced by reelin by analyzing one specific downstream effector (LIMK) and measuring the phosphorylation of its substrate cofilin [14], [58]. As observed in **Figure 11**, the total level of phospho-cofilin was significantly lower in neurons transfected with SNX17 shRNA. Overall, these results indicate that the availability of functional ApoER2 at the neuronal surface is controlled by SNX17, underscoring the role of this adaptor protein in the reelin signaling pathway.

## Discussion

In this paper, we uncovered an important role of SNX17 in ApoER2 trafficking and function. SNX17, by interacting with the ApoER2 cytoplasmic domain, facilitates receptor exit from a Rab5 to a Rab11 endosomal compartment and its recycling to the cell surface. These mechanisms influence ApoER2 cell surface levels and the reelin-induced signaling properties. In addition, we found that SNX17 participates in the reelin-induced receptor degradation pathway and regulates the processing of the carboxy-terminal fragment of ApoER2.

### Interaction of SNX17 with ApoER2

We first determined that the region responsible for the interaction with ApoER2 corresponded to amino acids 105 to 470 of SNX17, which were translated to to the F1, F2 and F3 subdomains of the SNX17 FERM domain and the C-terminal region [35]. This result is in agreement with previous observations, indicating that the FERM domain is responsible for the interaction with the APP protein [32]. The presence of the PX domain was not required for the *in vitro* interaction but was required for the function of SNX17 in the regulation of ApoER2 cell surface expression. Both the PX domain and the FERM domain, through binding to the cargo, are required for the recruitment of SNX17 to the endosomal membrane [35], [36]. Similarly, it has been shown for LRP1 and the scavenger receptor Stabilin1, that the region of 150–470 of SNX17 is the interacting domain [31], [59]. Using a yeast two-hybrid system, it was demonstrated that the minimal sequence of SNX17 able to interact with the LDL-R receptor is the region consisting of residues 113–389 [28], which strictly corresponds to the atypical FERM domain of SNX17 [35]. A similar region (FERM domain plus C-terminal region) was also proposed for the interaction of SNX17 with P-selectin, a protein that is not a member of the LDL-R superfamily [60]. It is interesting to note that within this region, specifically the F1 module was identified as the interacting domain with the GTPase H-RAS that is usually associated with the membrane and most likely links SNX17 to signaling processes [61], [35]. Altogether, this evidence indicates a conservative function for the FERM domain of SNX17 in the interaction with the counterpart receptors.

In addition, we determined that the multifunctional NPxY motif of ApoER2 is responsible for the interaction with SNX17.This falls in agreement with previous reports showing the interaction of SNX17 with the NPxY motif of LRP1, LDL-R, APP [28], [31], [32], and recently with β1-integrins [33], [34]. In the case of ApoER2, as was described for LRP1, the presence of negatively charged amino acids distal to the NPxY motif are most likely required for a stronger interaction with SNX17 [37]. The interaction between the receptor and adaptor protein could be abolished by phosphorylation of the tyrosine residue of the NPxY motif [62], as has been reported for LRP1 [63]. SNX17 also interacts with its partner when the tyrosine is replaced with a phenylalanine [59], as is the case for Stabilin1. Despite evidence supporting a role for the NPxY motif in the interaction of different partners with SNX17, Stockinger et al. [27] proposed that SNX17 does not interact with the NPxY motif of ApoER2. This result was first based on an indirect observation derived from a competitive ELISA assay using extracts of HEK293 cells transfected with SNX17 and purified Dab1 as a competitor. [27] As purified SNX17 was not relied upon in the assay, the cell extract of SNX17-transfected cells may have other proteins that could affect the activity of Dab1 as a competitor. In any case, our results demonstrate that ApoER2 interacts with the complete FERM domain- of SNX17 via the receptor's NPxY motif.

### SNX17 regulates the endosomal trafficking of ApoER2

SNX17 is an adaptor protein that is localized in the early endosome compartment [28], [30], [31], mostly due to the interaction of its PX domain with the characteristic early endosomal PI(3)P phospholipids [31], [60]. ApoER2 is constitutively internalized by a clathrin-mediated pathway [24]. Consistent with this evidence, we found that early after its internalization, ApoER2 and SNX17 colocalized in the endocytic route.

SNX17 has been related to different steps in endocytic trafficking, including increasing endocytosis, recycling, and/or affecting degradation of membrane proteins that bind to it [31]. In the case of ApoER2, we found that SNX17 does not participate in the initial steps of internalization but allows for more efficient recycling of the receptor to the plasma membrane in conditions without its ligand reelin. This effect was comparable in kidney and neuronal cell lines. In both cell types, the decrease in the ApoER2 recycling rate in the SNX17 knockdown cells was relatively constant compared with that of the control cells, suggesting a conserved contribution of SNX17 as part of the ApoER2 recycling machinery.

The knockdown of SNX17 resulted in significant retention of ApoER2 in the early endosomes after endocytosis, suggesting a role for SNX17 in the exit of ApoER2 from the early endosomal compartment. Accordingly, it has been shown that SNX17 localizes in the tubular domain of the early endosome, which is related to the recycling pathway [31]. The decrease that we observed in the colocalization of endocytosed ApoER2 and the recycling endosome marker Rab11 in SNX17 silenced cells suggests the participation of SNX17 in the transit of ApoER2 from early to recycling endosomes. However, we could not discard the possibility that SNX17 also controls the rapid recycling of the receptor directly from the early endosome to the plasma membrane. It is worth mentioning here that in contrast to the role in ApoER2 trafficking, SNX17 accomplishes a different role in the trafficking and degradation of integrin α5β1; by binding to the distal NPxY motif of the β1 subunit, SNX17 avoids integrin lysosomal degradation and aids in a more efficient recycling from the EEA1/Rab4-positive compartment. Very little α5β1 was found in Rab11 compartments in SNX17 knockdown cells, indicating that this adaptor protein regulates the trafficking of membrane proteins with different endosomal itineraries [34]. When the role of SNX17 in ApoER2 degradation was analyzed, we found unexpected results, considering what has been demonstrated for LRP1 [31] and integrins [34]. In the SNX17 knockdown cells, ApoER2 was not found at higher levels in the late endosomes (**Figure 4**) nor was more ApoER2 degraded (**Figure 5**), suggesting that the pool of constitutively endocytosed ApoER2 requires a signal other than early endosome retention to be degraded. In contrast, when ApoER2 was stimulated with reelin, the absence of SNX17 was associated with even lower levels of the receptor, implying that under normal conditions, SNX17 prevents the degradation of the signaling receptor stimulated by its ligand and that is possibly induced by receptor ubiquitination. Although ApoER2 ubiquitination has not been directly demonstrated, ApoER2 co-expression with the E3 ubiquitin ligase IDOL and/or the activation of the nuclear receptor LXR that induces IDOL expression [64], decreased the total levels of the receptor suggesting that this post-translational modification could regulate ApoER2 half-life [43], [65].

The role of SNX17 in ApoER2 recycling is dependent on the specific interaction with the receptor and is not caused by a general effect on the endosomal compartment because SNX17 knockdown did not affect the recycling of megalin/LRP2 [27]. Our results are in agreement with previous observations showing that SNX17 facilitates the recycling of LRP1 [31]. Other results also indirectly relate SNX17 with promoting the recycling and preventing the degradation of its partners. In the case of LDL-R, SNX17 overexpression increases the number of endocytic cycles, implying an increase in recycling efficiency [27]. In the case of P-selectin, the overexpression of SNX17 concomitant with the increase in the receptor endocytosis, increases its recycling [30] while avoiding entry into the multivesicular bodies/late endosomes [60]. As was mentioned, an active role of SNX17 was recently shown in the stimulation of integrin recycling [33], [34]. It has also been shown that SNX17 silencing decreases the surface levels of APP, which is associated with deficient recycling [32].

### ApoER2 processing and SNX17

In addition to Dab1, a number of adaptors proteins, including Dab2, Fe65, and X11α/Mint, bind to the ApoER2 cytoplasmic domain and regulate its trafficking and signaling [38]. Interestingly, some of these proteins also regulate the proteolytic processing of this receptor [42], [54], [55], [66] by binding to its NPxY motif. For example, Dab1 overexpression increases the surface levels of ApoER2 and APP, along with an increase in the processing of both proteins [54]. In addition, the α-secretase inhibitor TIMP3 decreases the processing of ApoER2 [41], and there is evidence indicating that the activity of α-secretase is present at the plasma membrane [67]. Altogether, these facts suggest that the location where the first cleavage of ApoER2 occurs is the plasma membrane, at least under unstimulated conditions. Unexpectedly, we observed that under SNX17 silencing conditions in which, at steady state, the receptor is less expressed at the cell surface and accumulates in a Rab5-positive early endosome, there was an increase in the ApoER2 CTF level. Taking this result and the other previously published evidence into account, we hypothesize that the accumulation of CTF in SNX17 knockdown cells is most likely not due to an increase in the α-secretase processing step but is likely related to a reduction the second cleavage step, which is catalyzed by the γ-secretase complex. The γ-secretase activity was not altered in cells with different expression levels of SNX17 (control vs.knockdown SNX17 cells). However, inhibition of the γ-secretase complex resulted in an increase in the CTF level only in control cells, indicating that SNX17 normally facilitates the cleavage step catalyzed by this complex. It is not clear why this processing may be impeded in SNX17 silenced cells. Different studies have analyzed the location of the endogenous mature γ-secretase, concluding that it is situated in the TGN and/or the endosomal pathway. In N2a cells, γ-secretase co-localizes in TGN vesicles with the markers syntaxin 6 and VAMP4 and in the late endosomal membranes, labeled with syntaxin 13 [68]. Hence, it was also determined that a membrane fraction enriched in syntaxin 13 has more γ-secretase activity in rat brain extracts [69]. Syntaxin 13 resides in tubular structures that connect the early and recycling endosomes and have been suggested to mediate protein recycling via early/recycling tubulovesicular endosomes that are enriched in Rab11 endosomes [70], [71]. In agreement with this evidence, it has been shown that the mature cleaved form of presenilin1 (active component of γ-secretase) is present in fractions enriched with the recycling endosomal marker transferring receptor (TfnR) after subcellular fractionation, and this enrichment is correlated with high levels of colocalization of these two proteins [72]. Moreover, presenilin1 interacts with the recycling endosome protein Rab11 [73]. In this scenario, it is possible to propose that SNX17 knockdown induces the retention of ApoER2-CTF in early endosomes, affecting its encounter with and processing by γ-secretase in recycling and/or late endosomes.

### SNX17 and reelin signaling pathway

Signaling receptors can be degraded or recycled to the surface after binding of their ligands [74] and can be active at the surface and/or intracellularly. Therefore, the endocytosis/recycling pathways are important for regulating the signaling strength. Reelin induces receptor degradation and proteolysis under normal conditions [42], [43], and SNX17 controls both processes (**Figure 6** and **Figure 7**).

To specifically evaluate the role of SNX17 in the reelin signaling pathway, SNX17 was silenced in hippocampal and cortical neurons. Reelin has essential roles in neuronal development, in the regulation of synaptic function and in neuronal survival [75]. These effects are related to reelin's ability to interact with ApoER2 and VLDLR, inducing their clustering [76], which results in Dab1 phosphorylation and the activation of different effectors [52], [77], [78]. In neurons with lower levels of cell surface ApoER2, resulting from SNX17 downregulation, an evident decrease in reelin-mediated Dab1, AKT, and GSK3β phosphorylation was observed. Because VLDLR is also able to interact with SNX17 [27] as determined by GST pull-down, we cannot rule out a possible effect of SNX17 knockdown in VLDLR trafficking and signaling. In fact, it has been shown that knockout of both ApoER2 and VLDLR proteins is required to prevent reelin-induced phosphorylation of Dab1 [52], suggesting that both proteins could be altered in SNX17 knockdown cells. In our studies, however, we know that ApoER2 is involved in the effects produced by the decrease in SNX17 because the activation of the Dab1-LIMK axis, as detected by cofilin phosphorylation at serine 3, was significantly reduced in silenced neurons treated with reelin.

Finally, we also determined that SNX17 alters the most common effect induced by long-term reelin treatment, which is the increase in the dendritic arborization [20]. Under silencing conditions, the dendritic outgrowth was not affected, with the exception of the number of secondary dendrites. However, upon long-term incubation with reelin, the lack of SNX17 reduced the effect of this ligand, although not to the level of the control cells not treated with reelin.

Until now, we have centered the discussion of the neuronal roles of SNX17 and the consequences of its decrease on its effect on reelin signaling receptors, especially ApoER2. However, it is important to note that other signaling pathways could be regulated by SNX17, considering its other receptor partners. The lipoprotein receptor LRP1 has neuronal functions and has been shown to be involved in transmitter-dependent post-synaptic responses caused by its interaction with the post-synaptic proteins PSD-95 and NMDA [79]. Additionally it has been shown that the interaction of APP with SNX17 regulates its processing [32]. Moreover, APP also increases dendritic outgrowth in the presence of reelin [80]. Therefore, this adaptor protein could play an important function in neuronal physiology, especially when the role of SNX17 in the trafficking of lipoprotein receptors and APP is considered.

In summary, our results show that SNX17, by interacting with the NPxY domain of ApoER2, facilitates its trafficking from early endosomes to recycling endosomes and to the plasma membrane. As the receptor CTF is, in principle, also able to interact with SNX17 this could explain the effect of SNX17 silencing in CTF accumulation. Interestingly, the role of SNX17 in receptor half-life would be dependent on the endocytosis “mode” of ApoER2, protecting the receptor from degradation when the receptor is engaged in reelin internalization. Therefore, the presence of SNX17 would be necessary for an optimal ApoER2/reelin-mediated signaling activation in neurons and may function, by positively regulating the cell surface level of the receptor.

## Supporting Information

Figure S1 Expression levels of endogenous and transfected myc-SNX17 in control and SNX17 silenced cells. In order to recover the cell surface expression of ApoER2 reduced by silencing SNX17, control (pLKO) or SNX17 knockdown HEK293 cells were co-transfected with ApoER2 and either with pCDNA3 (control, -), or myc-SNX17 (+) from mouse. To detect the expression levels of SNX17, the cells were lysed and the presence of the adaptor protein was visualized by western blot with a rabbit polyclonal anti SNX17 antibody. (**A**) Levels of SNX17 from one representative experiment corresponding to Figure 2F, in which the surface/total level of ApoER2 was determined. (**B,C**) In addition to the role of the full length SNX17, the ability to recover the phenotype was determined for different deletion constructs, SNX17-2-250 corresponding to a deletion of the F3 subdomain (that does not bind ApoER2 in GST pull down assay) and the SNX17 105–470, lacking the PX domain (that binds to ApoER2 in the GST-pull down assay) (Figure 1); the presence of SNX17 was determined with anti-myc. (**D**) Levels of SNX17 from one representative experiment corresponding to Figure 6D, E, in which the role of SNX17 in the levels of ApoER2-CTF was determined.
(TIF)

Figure S2 SNX17 knockdown does not alter ApoER2 arrival to the early endosome. HeLa pLKO and SNX17 silenced clones were transfected with HA-ApoER2, RAP, and GFP-Rab5. Cells were incubated with anti-HA antibody for 1 h at 4°C and then shifted to 37°C for 10 min to allow for receptor internalization. After this period of time, the antibody remaining at the surface was removed by acid wash. Cells were washed, permeabilized, and incubated with Alexa 594-conjugated goat anti-mouse IgG. Images were captured by confocal microscopy, and Mander's colocalization index and Pearson's coefficient were calculated in 10 cells for each condition. Bars, 10 μm.
(TIF)

Figure S3 The activity of γ-secretase is not modified in cells with reduced levels of SNX17. Control (pLKO) or SNX17 knockdown N2a cells expressing ApoER2 were lysed in CHAPSO buffer. Measurement of γ-secretase activity was performed using a fluorogenic substrate assay, which is based on the secretase-dependent cleavage of a -secretase-specific substrate conjugated with a fluorescent molecule.
(TIF)

Figure S4 SNX17 knockdown in neurons. Mouse dissociated cortical neurons were transfected at DIV 5 with GFP and the corresponding shRNA plasmid. After 48 h, cells were fixed and analyzed by immunofluorescence using an anti-SNX17 antibody. The figure shows that when cells are positive for GFP, they are also negative for SNX17 in the neurons transfected with SNX17 shRNA.
(TIF)

Figure S5 SNX17 knockdown alters the number and length of dendrites induced by reelin. Mouse dissociated hippocampal neurons were transfected with GFP expression plasmid and the corresponding shRNA, plasmid. After three days, the neurons were treated with reelin for 3 days, fixed, and analyzed by immunofluorescence. Images were captured by confocal microscopy. Quantitative analysis of the length and number of primary and secondary dendrites was performed by making individual tracings and using the Neuron J plugin. The lengths of primary and secondary neurites were significantly reduced upon reelin treatment in SNX17 knockdown neurons, whereas only secondary neurites were reduced in number in the silenced neurons. *p<0.05; **p<0.01.
(TIF)

Methods S1 SNX17 silencing in neurons. A total of 1×10^5^ mouse dissociated cortical neurons were transfected at DIV 4 with GFP and the corresponding shRNA plasmid (0.3 μg each) using Lipofectamine 2000. After 3 days, the cells were fixed with 4% PFA and 4% sucrose for 20 min and processed for immunofluorescence with a rabbit anti-SNX17 (1∶250). Later cells were stained with an Alexa 555-conjugated anti-rabbit antibody. Images of individual cells were captured with an inverted LSM 510 Zeiss microscope with a 63 X oil immersion lens, and images were analyzed using ImageJ software.
(DOCX)
