# Supplementary figures and images for: Sorting Nexin 17 Regulates ApoER2 Recycling and Reelin Signaling
Source: PLoS One. 2014 Apr 4;9(4):e93672. doi: 10.1371/journal.pone.0093672 (PMC3976305; doi:10.1371/journal.pone.0093672)

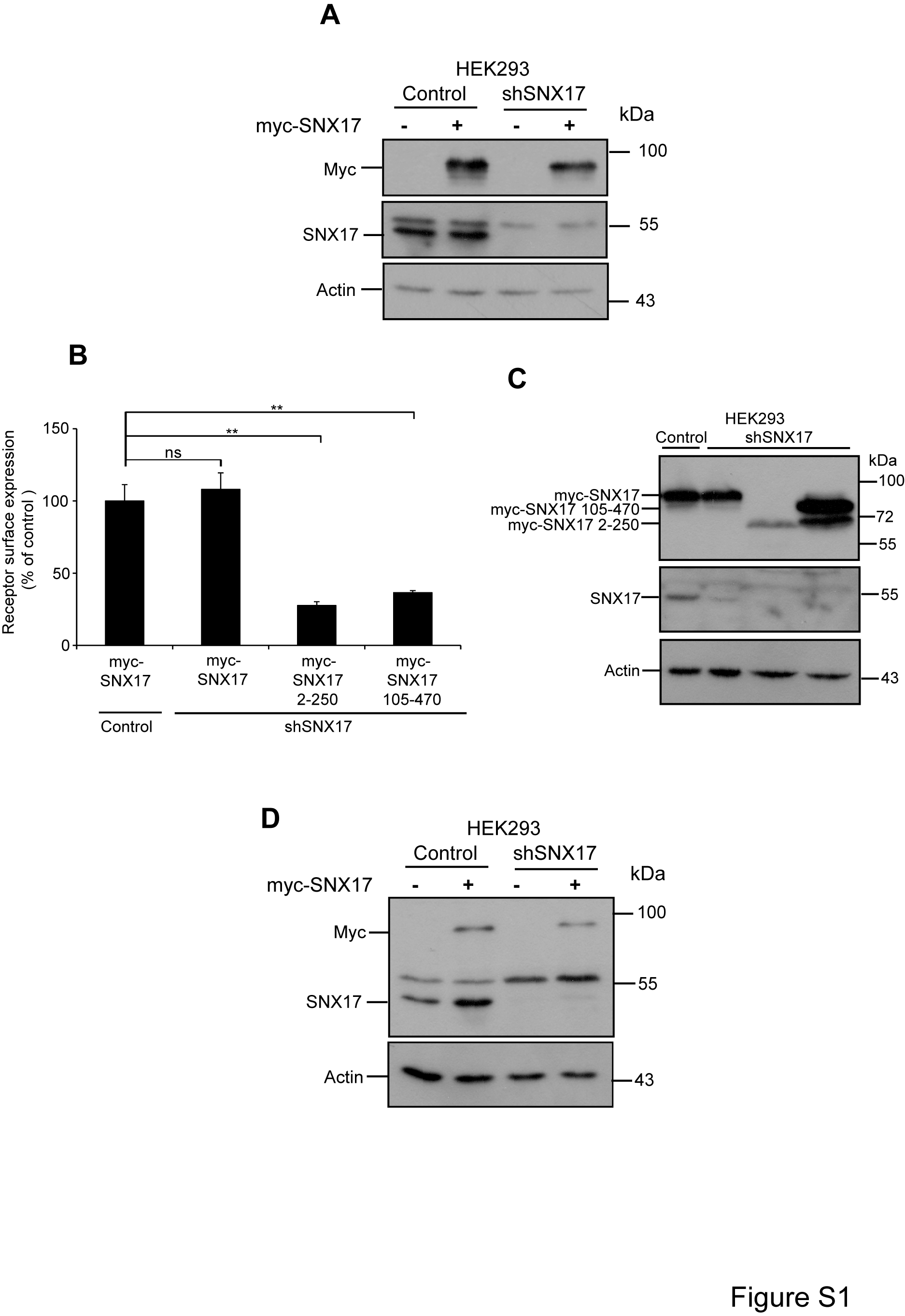

Supplement: Figure S1 — Expression levels of endogenous and transfected myc-SNX17 in control and SNX17 silenced cells. In order to recover the cell surface expression of ApoER2 reduced by silencing SNX17, control (pLKO) or SNX17 knockdown HEK293 cells were co-transfected with ApoER2 and either with pCDNA3 (control, -), or myc-SNX17 (+) from mouse. To detect the expression levels of SNX17, the cells were lysed and the presence of the adaptor protein was visualized by western blot with a rabbit polyclonal anti SNX17 antibody. (A) Levels of SNX17 from one representative experiment corresponding to Figure 2F, in which the surface/total level of ApoER2 was determined. (B,C) In addition to the role of the full length SNX17, the ability to recover the phenotype was determined for different deletion constructs, SNX17-2-250 corresponding to a deletion of the F3 subdomain (that does not bind ApoER2 in GST pull down assay) and the SNX17 105–470, lacking the PX domain (that binds to ApoER2 in the GST-pull down assay) (Figure 1); the presence of SNX17 was determined with anti-myc. (D) Levels of SNX17 from one representative experiment corresponding to Figure 6D, E, in which the role of SNX17 in the levels of ApoER2-CTF was determined. (TIF) [file pone.0093672.s001.tif]

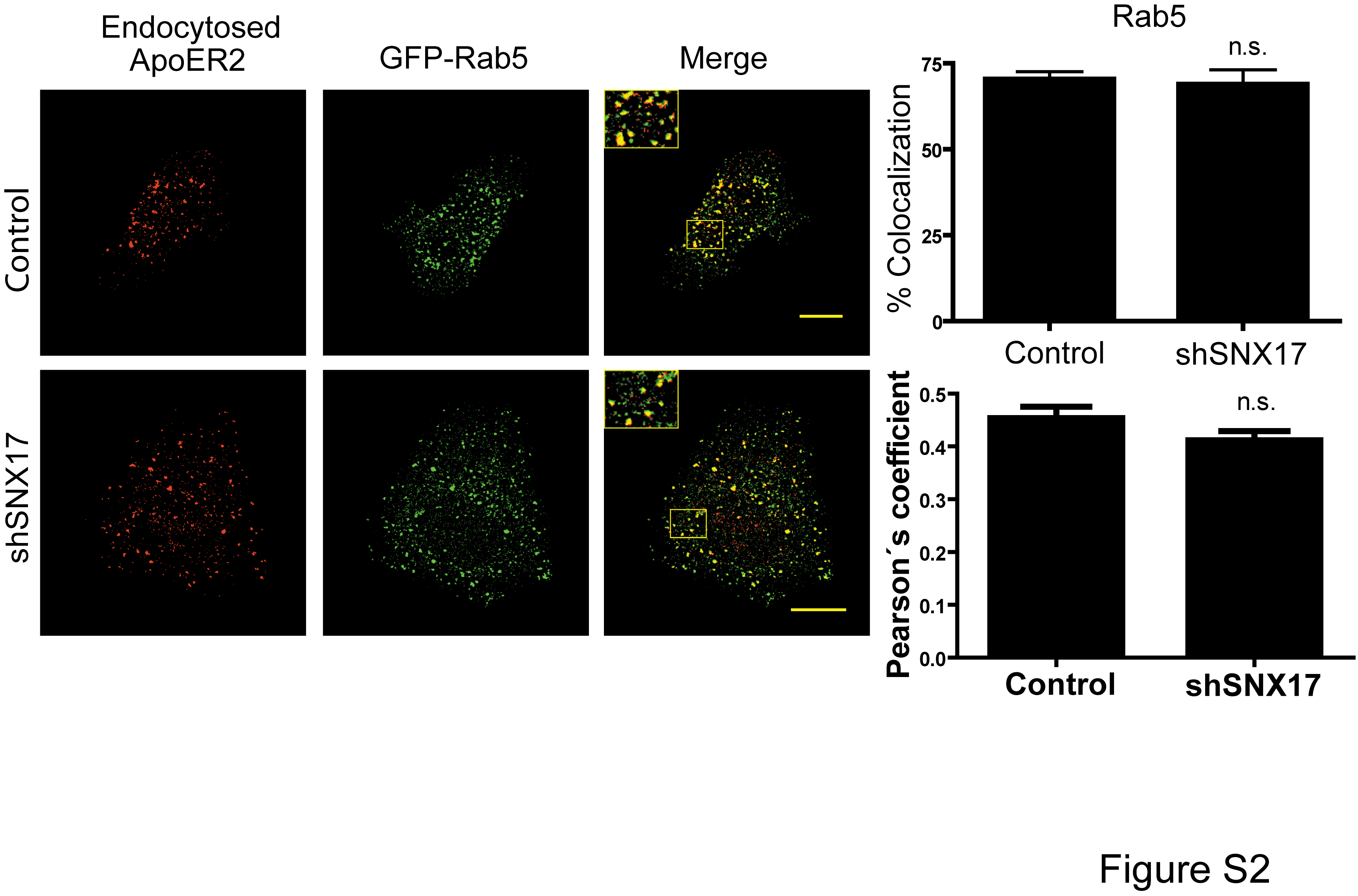

Supplement: Figure S2 — SNX17 knockdown does not alter ApoER2 arrival to the early endosome. HeLa pLKO and SNX17 silenced clones were transfected with HA-ApoER2, RAP, and GFP-Rab5. Cells were incubated with anti-HA antibody for 1 h at 4°C and then shifted to 37°C for 10 min to allow for receptor internalization. After this period of time, the antibody remaining at the surface was removed by acid wash. Cells were washed, permeabilized, and incubated with Alexa 594-conjugated goat anti-mouse IgG. Images were captured by confocal microscopy, and Mander's colocalization index and Pearson's coefficient were calculated in 10 cells for each condition. Bars, 10 μm. (TIF) [file pone.0093672.s002.tif]

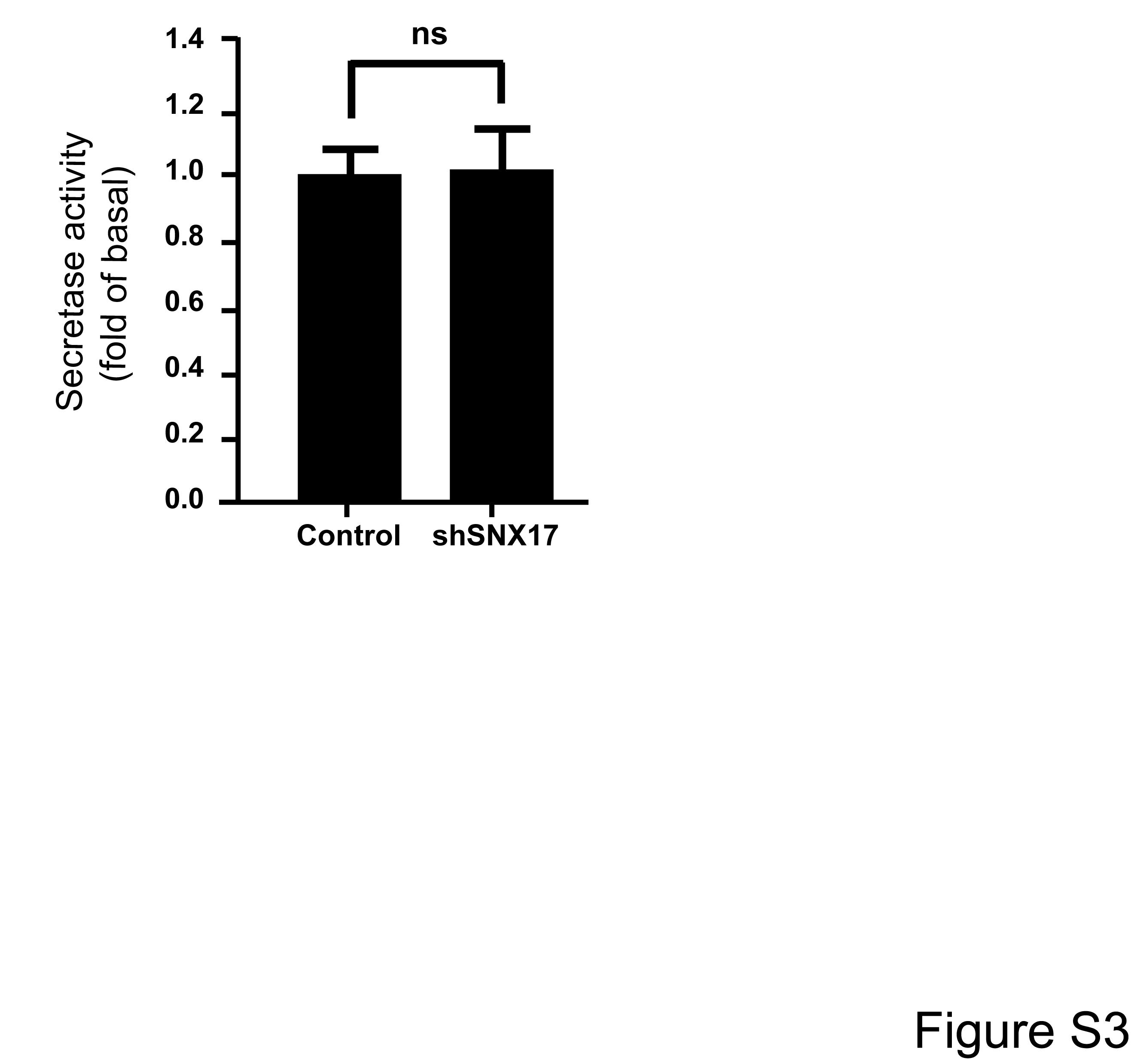

Supplement: Figure S3 — The activity of γ-secretase is not modified in cells with reduced levels of SNX17. Control (pLKO) or SNX17 knockdown N2a cells expressing ApoER2 were lysed in CHAPSO buffer. Measurement of γ-secretase activity was performed using a fluorogenic substrate assay, which is based on the secretase-dependent cleavage of a -secretase-specific substrate conjugated with a fluorescent molecule. (TIF) [file pone.0093672.s003.tif]

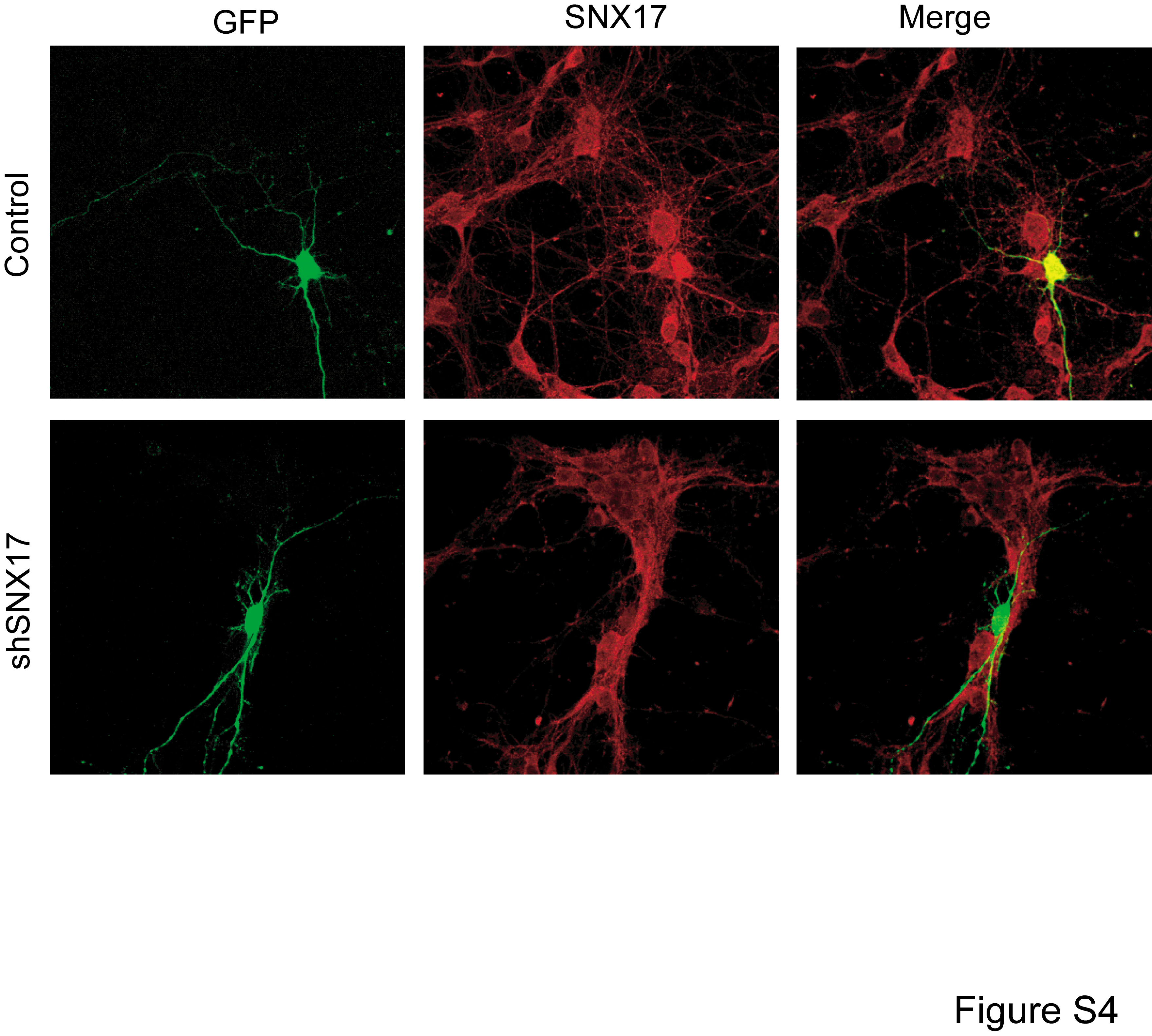

Supplement: Figure S4 — SNX17 knockdown in neurons. Mouse dissociated cortical neurons were transfected at DIV 5 with GFP and the corresponding shRNA plasmid. After 48 h, cells were fixed and analyzed by immunofluorescence using an anti-SNX17 antibody. The figure shows that when cells are positive for GFP, they are also negative for SNX17 in the neurons transfected with SNX17 shRNA. (TIF) [file pone.0093672.s004.tif]

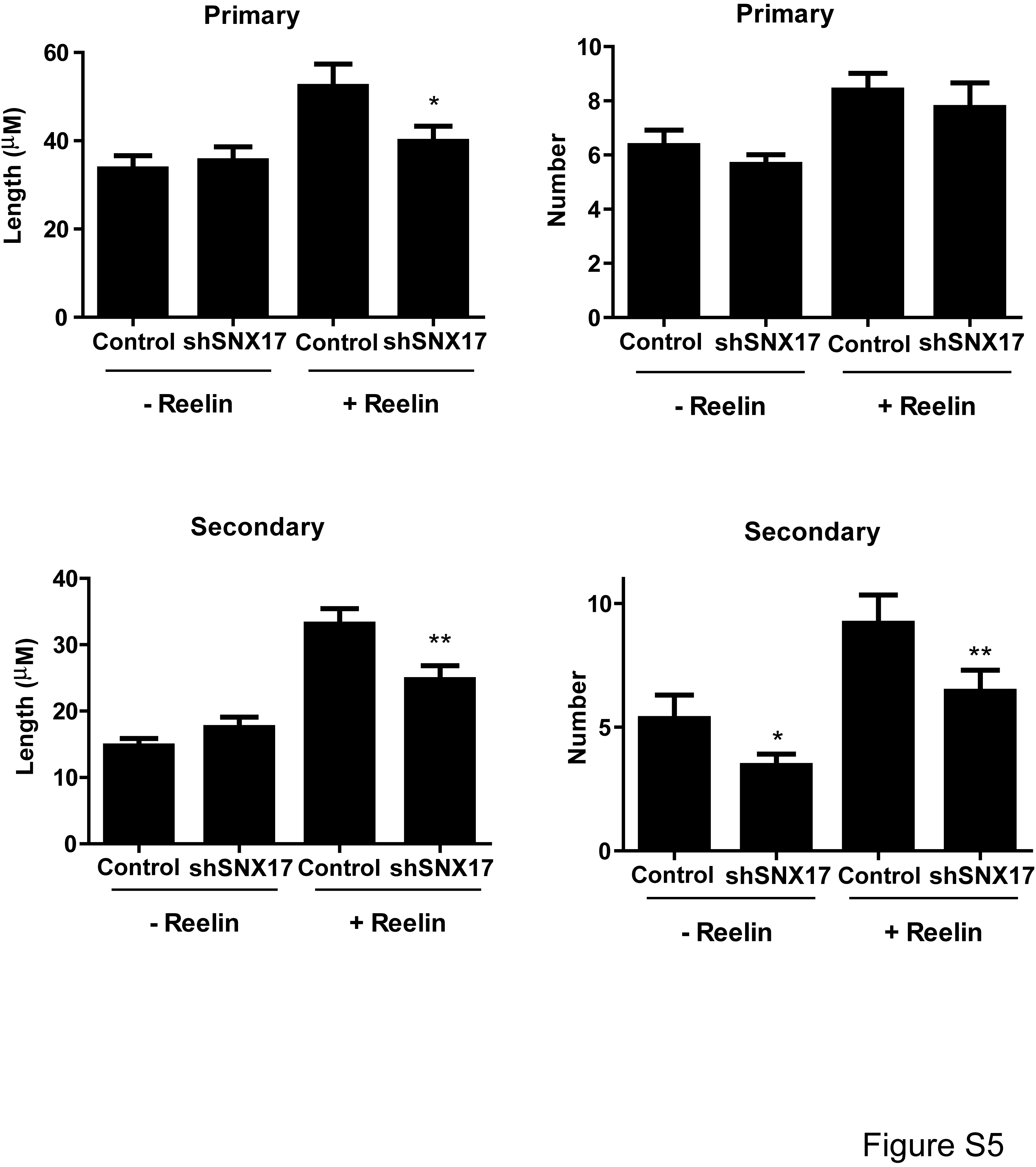

Supplement: Figure S5 — SNX17 knockdown alters the number and length of dendrites induced by reelin. Mouse dissociated hippocampal neurons were transfected with GFP expression plasmid and the corresponding shRNA, plasmid. After three days, the neurons were treated with reelin for 3 days, fixed, and analyzed by immunofluorescence. Images were captured by confocal microscopy. Quantitative analysis of the length and number of primary and secondary dendrites was performed by making individual tracings and using the Neuron J plugin. The lengths of primary and secondary neurites were significantly reduced upon reelin treatment in SNX17 knockdown neurons, whereas only secondary neurites were reduced in number in the silenced neurons. *p<0.05; **p<0.01. (TIF) [file pone.0093672.s005.tif]
