# Supplementary material for: Sorting Nexin 17 Regulates ApoER2 Recycling and Reelin Signaling
Source: PLoS One. 2014 Apr 4;9(4):e93672. doi: 10.1371/journal.pone.0093672 (PMC3976305; doi:10.1371/journal.pone.0093672)
Supplement: Methods S1 — SNX17 silencing in neurons. A total of 1×10^5^ mouse dissociated cortical neurons were transfected at DIV 4 with GFP and the corresponding shRNA plasmid (0.3 μg each) using Lipofectamine 2000. After 3 days, the cells were fixed with 4% PFA and 4% sucrose for 20 min and processed for immunofluorescence with a rabbit anti-SNX17 (1∶250). Later cells were stained with an Alexa 555-conjugated anti-rabbit antibody. Images of individual cells were captured with an inverted LSM 510 Zeiss microscope with a 63 X oil immersion lens, and images were analyzed using ImageJ software. (DOCX) [file pone.0093672.s006.docx]

**Supplementary Methods**

*SNX17 silencing in neurons.*

A total of 1x10^5^ mouse dissociated cortical neurons were transfected at DIV 4 with GFP and the corresponding shRNA plasmid (0.3 µg each) using Lipofectamine 2000. After 3 days, the cells were fixed with 4% PFA and 4% sucrose for 20 min and processed for immunofluorescence with a rabbit anti-SNX17 (1:250). Later cells were stained with an Alexa 555-conjugated anti-rabbit antibody. Images of individual cells were captured with an inverted LSM 510 Zeiss microscope with a 63 X oil immersion lens, and images were analyzed using ImageJ software.
